# Single cell sequencing of radial glia progeny reveals the diversity of newborn neurons in the adult zebrafish brain

**DOI:** 10.1242/dev.185595

**Published:** 2020-01-09

**Authors:** Christian Lange, Fabian Rost, Anja Machate, Susanne Reinhardt, Matthias Lesche, Anke Weber, Veronika Kuscha, Andreas Dahl, Steffen Rulands, Michael Brand

**Affiliations:** 1 Center for Regenerative Therapies Dresden (CRTD), CMCB, Technische Universität Dresden, Fetscherstrasse 105, 01307 Dresden, Germany; 2 Max Planck Institute for the Physics of Complex Systems, Noethnitzer Strasse 38, 01187 Dresden, Germany; 3 Center for Systems Biology Dresden (CSBD), Pfotenhauer Strasse 108, 01307 Dresden, Germany; 4 DRESDEN-concept Genome Center, c/o Center for Molecular and Cellular Bioengineering (CMCB), Technische Universität Dresden, Fetscherstrasse 105, 01307, Dresden, Germany

**Keywords:** Adult neurogenesis, Zebrafish, Radial glia, Neural stem cell, Telencephalon, Single cell sequencing

## Abstract

Zebrafish display widespread and pronounced adult neurogenesis, which is fundamental for their regeneration capability after central nervous system injury. However, the cellular identity and the biological properties of adult newborn neurons are elusive for most brain areas. Here, we have used short-term lineage tracing of radial glia progeny to prospectively isolate newborn neurons from the her4.1^+^ radial glia lineage in the homeostatic adult forebrain. Transcriptome analysis of radial glia, newborn neurons and mature neurons using single cell sequencing identified distinct transcriptional profiles, including novel markers for each population. Specifically, we detected two separate newborn neuron types, which showed diversity of cell fate commitment and location. Further analyses showed that these cell types are homologous to neurogenic cells in the mammalian brain, identified neurogenic commitment in proliferating radial glia and indicated that glutamatergic projection neurons are generated in the adult zebrafish telencephalon. Thus, we prospectively isolated adult newborn neurons from the adult zebrafish forebrain, identified markers for newborn and mature neurons in the adult brain, and revealed intrinsic heterogeneity among adult newborn neurons and their homology with mammalian adult neurogenic cell types.

## Abstract (precis)

**Summary:** A combination of lineage tracing and single cell sequencing identifies specific markers and functional subpopulations of adult newborn neurons in the zebrafish forebrain.

## INTRODUCTION

Adult neurogenesis, the generation and integration of additional neurons to the brain circuitry in adulthood, is widespread across vertebrates, although the extent greatly varies among species (Kaslin et al., 2008; Grandel and Brand, 2013; Alunni and Bally-Cuif, 2016). It is thought to support learning and memory (Aimone et al., 2014; Frisén, 2016), but can also function to replace neurons that are lost due to disease or injury in some species (Reimer et al., 2008; Berg et al., 2010; Kroehne et al., 2011; März et al., 2011; Baumgart et al., 2012; Amamoto et al., 2016; Kaslin et al., 2017).

In most mammals, adult neurogenesis is confined to the major neurogenic niches in the subgranular zone of the hippocampal dentate gyrus and the sub-ependymal zone of the lateral ventricles. Newborn neurons (NBNs) integrate only into the granular zone of the dentate gyrus, and into the olfactory bulb in rodents or into the striatum in humans (Aimone et al., 2014; Ernst and Frisén, 2015). Outside these target zones for adult neurogenesis, integration of NBNs is essentially absent or minimal, and most brain areas (including the cerebral cortex) show limited potential for NBN integration and survival, even after injury-induced neuronal cell death (Bhardwaj et al., 2006; Ohab et al., 2006; Saha et al., 2013; Huttner et al., 2014). Thus, a low potential for NBN integration in most brain areas represents an important roadblock for brain repair after injury or neurodegeneration. To develop therapeutic strategies for cell replacement, a better understanding of the mechanisms that might allow neurogenesis and neuron integration in such brain areas is needed.

In contrast to mammals, teleost fish display widespread and pronounced adult neurogenesis (Ekström et al., 2001; Zupanc et al., 2005; Adolf et al., 2006; Grandel et al., 2006; Alunni et al., 2010; Ganz and Brand, 2016). In the adult zebrafish brain, we previously identified 17 different proliferative zones throughout the neuraxis. These proliferative zones are accompanied by adjacent zones, where newborn neurons integrate. In the telencephalon, the cell bodies of radial glia (RG) neural stem cells are localized in the ventricular zone (VZ), but they span the entire telencephalon with their processes (Grandel et al., 2006; Adolf et al., 2006; Ganz et al., 2010). In the dorsal telencephalon (pallium), RG divide constitutively in adulthood and give rise to neurons, which undergo short-distance migration to the adjacent peri-ventricular zone (PVZ) of the parenchyma, where they are added to the circuitry (Ganz et al., 2010; Kroehne et al., 2011; Furlan et al., 2017). Cells in the PVZ express markers of glutamatergic projection neuron development, such as *tbr1*, *neurod1* and *bhlhe22* (Ganz et al., 2012; Furlan et al., 2017). Similar to the developing mammalian forebrain, a second population of neural progenitors, expressing the marker nestin, exists in the VZ of the striatal ventral telencephalon, which expresses markers of GABAergic interneuron progenitors, e.g. *dlx2* and *dlx5a* (März et al., 2010a; Ganz et al., 2012). The ventrally generated neurons undergo long-distance migration into the telencephalic parenchyma, reminiscent of interneuron tangential migration in mammalian development (Ganz et al., 2010). These data indicate that, in zebrafish telencephalon, the dorsal pallium and the ventral striatum – corresponding to the cognate mammalian brain territories – display ongoing neurogenesis and NBN integration in an evolutionarily conserved manner.

In contrast to mammals, zebrafish efficiently repair lesions after injury to the telencephalon through induction of (1) proliferation of radial glia, (2) neuron generation and (3) integration of newborn, differentiated neurons in the parenchyma (Kroehne et al., 2009, 2011; Baumgart et al., 2012; März et al., 2011; Skaggs et al., 2014). Within weeks and months of the injury, the lesion site is dramatically reduced in size and neuronal connections in the lesioned hemisphere, which are initially destroyed, re-appear. Lineage tracing shows that these regenerated neurons derive from RG and persist long term (Kroehne et al., 2011). The molecular mechanisms that enable this repair process are currently incompletely understood. In particular, previous research focused on the regulation of RG as the source of NBNs in homeostasis or after injury, while the role of immature neuronally committed progenitor cells and neurons, at various stages of their maturation and integration into the adult telencephalon, remains poorly understood.

Recently, cellular differentiation trajectories were reconstructed using single cell sequencing – alone or in combination with cellular barcoding – in vertebrate embryos (Alemany et al., 2018; Briggs et al., 2018; Farrell et al., 2018; Spanjaard et al., 2018; Wagner et al., 2018) or in the zebrafish juvenile brain (Raj et al., 2018). However, neurogenesis and NBN differentiation in the adult telencephalon has not been investigated using these methods. To gain insight into the role and regulation of NBNs in adult neurogenesis in the zebrafish forebrain, we devised a strategy to lineage trace RG, RG-derived NBNs and MNs, allowing their direct, specific isolation from heterogenous cell populations (i.e. prospective isolation). Transcriptome analysis by single cell sequencing revealed pronounced heterogeneity among RG-derived NBNs and allowed the analysis of differentiation trajectories in the adult zebrafish forebrain.

## RESULTS

### Lineage tracing of radial glia-derived newborn neurons in the adult zebrafish telencephalon

**Fig. 1.**
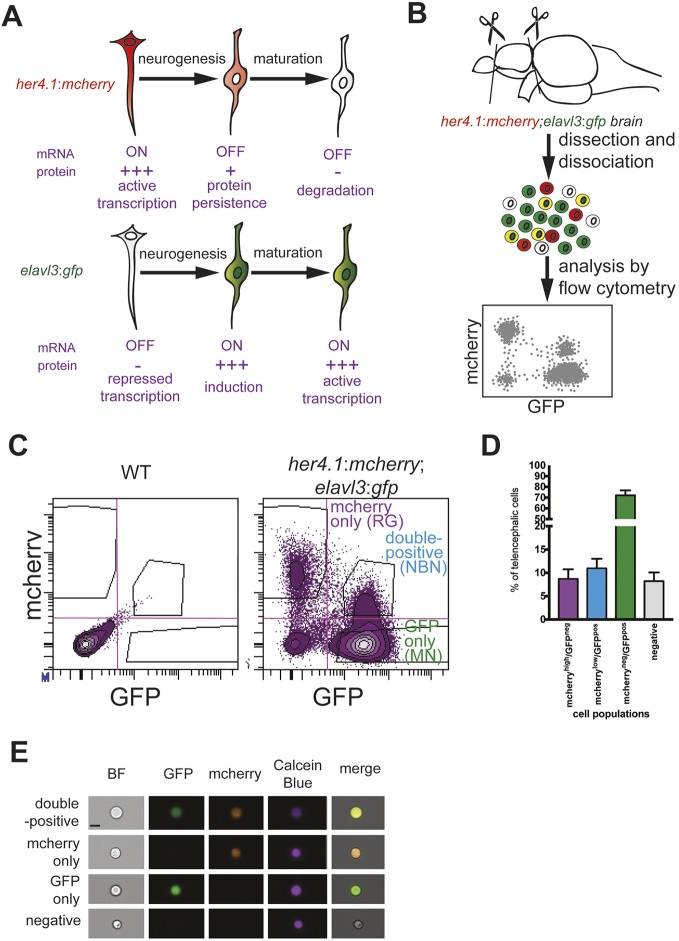
**A double-reporter line for identification and quantification of radial glia-derived newborn neurons in the adult zebrafish brain.** (A) Model for the lineage-trace paradigm. NBNs stop to transcribe *her4.1*-driven *mcherry*, but inherit the protein from their RG precursors (top). At the same time, NBNs induce transcription of the neuronal marker *elavl3*:*gfp* (bottom), allowing the identification of NBNs as mCherry/GFP-double-positive cells in double reporter fish. (B) Scheme of the analytical workflow. Forebrains are removed from double reporter fish and dissociated, and cell types are analyzed by flow cytometry. (C) Representative FACS plots showing forebrain cells from wild-type (left) and *her4.1*:*mcherry*;*elavl3*:*gfp* double reporter fish (right). There are clearly separated populations of mCherry^high^/GFP^neg^ RG and mCherry^low^/GFP^pos^ NBNs. (D) Quantification of cells in the four quadrants shown in C, revealing that NBNs can be found in a frequency comparable with RG and represent circa 10% of forebrain cells. *n*=5. Data are mean±s.e.m. (E) Representative single cells imaged in flow cytometry for mCherry^pos^/GFP^pos^ (first row), mCherry^pos^/GFP^neg^ (second row), mCherry^neg^/GFP^pos^ (third row) and mCherry^neg^/GFP^neg^ (fourth row). At least 20 cells were analyzed and no doublets were seen, excluding the double-positive cells that represent doublets. Scale bar: 7 µm.

In order to prospectively isolate the neuronal progeny of radial glia (i.e. NBNs) in the adult zebrafish telencephalon, we developed a short-term lineage-tracing protocol, based on retention of fluorescent proteins in cell type-specific, fluorescent reporter lines. To this end, we combined the neuronal reporter line *elavl3:gfp* (Park et al., 2000) with the *her4.1:mcherry* reporter line that marks RG (Kroehne et al., 2011). Although the expression of *mcherry* mRNA under the control of the her4.1 promotor is restricted to radial glia and rapidly downregulated in NBN, fluorescent proteins, which have a half-life of circa 24 h (Li et al., 1998), are inherited by the neuronal daughters of dividing radial glia in detectable amounts (Furlan et al., 2017). Using this approach, newborn neurons could be identified as mCherry/GFP double-positive cells in the telencephalon of *her4.1:mcherry*; *elavl3:gfp* fish (Fig. 1A). When analyzing dissociated cells from *her4.1:mcherry*; *elavl3:gfp* forebrains, consisting of the telencephalon and anterior diencephalon (see Fig. 1B and Materials and Methods) with flow cytometry, we found that cells with high levels of mCherry were GFP negative. In contrast, cells with low, but detectable levels of mCherry were clearly positive for the neuronal marker *elavl3*:GFP (Fig. 1C). These results confirmed our hypothesis that NBNs can be identified as mCherry/GFP double-positive cells in the telencephalon of *her4.1:mcherry*;*elavl3:gfp* fish, while bona fide RG had higher average levels of mCherry, but were GFP negative. Forebrain cells from single-transgenic *her4.1:mcherry* or *elavl3:gfp* fish contained only few mCherry and GFP double-positive cells, which are likely autofluorescent cells (Fig. S1A). Quantification showed that mCherry^high^/GFP^neg^ RG represented 8.7%, whereas mCherry^low^/GFP^pos^ cells (designated as NBNs) accounted for 10.9% of all live forebrain cells. Mature neurons (MN; mCherry^neg^/GFP^pos^) were the dominating cell population in the adult forebrain with 72.1%, and lineage marker-negative cells accounted for 8.2% of forebrain cells (Fig. 1D). Direct imaging of dissociated cells by Image Stream flow cytometry confirmed the presence of single cells that are simultaneously positive for mCherry and GFP, excluding the alternative possibility that these cells might have represented doublets of cells expressing either GFP or mCherry (Fig. 1E).

**Fig. 2.**
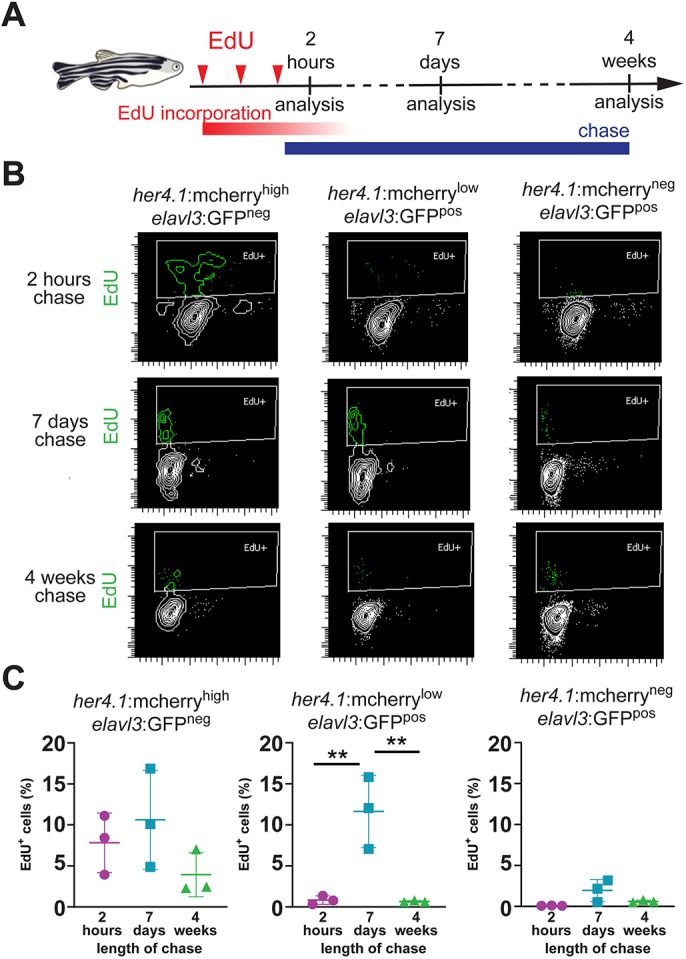
**Newborn neurons retain the radial glia-derived reporter signal transiently.** (A) Scheme of experimental design, indicating the timing of EdU injections (red arrowheads), EdU incorporation (red bar), EdU chase (blue bar) and analysis timepoints after the last EdU injection. (B) Flow cytometry plots showing EdU (green) incorporation in sorted *her4.1*:mCherry^high^/*elavl3*:GFP^neg^ cells (left), *her4.1*:mCherry^low^/*elavl3*:GFP^pos^ cells (middle) and *her4.1*:mCherry^neg^/*elavl3*:GFP^pos^ cells (right) after 2 h (top), 7 days (middle) or 4 weeks (bottom) of chase. There is robust EdU labeling in *her4.1*:mCherry^low^/*elavl3*:GFP^pos^ after 7 days, but not after 2 h or 4 weeks chase. (C) Quantification of EdU labeling *her4.1*:mCherry^high^/*elavl3*:GFP^neg^ cells (left), *her4.1*:mCherry^low^/*elavl3*:GFP^pos^ cells (middle) and *her4.1*:mCherry^neg^/*elavl3*:GFP^pos^ cells (right) after 2 h (magenta), 7 days (blue) or 4 weeks (green). Single data points are shown; *n*=3; data are mean±s.e.m. **P<0.01 by one-way ANOVA and Bonferroni's *post hoc* test.

As her4.1 is a main target gene of the Notch pathway, mCherry^low^/GFP^pos^ cells could also represent mature neurons with low level activation of this pathway instead of being NBNs that inherited mCherry protein from their RG precursors. To exclude this possibility, we combined the *elavl3:gfp* neuronal reporter line with a newly generated radial glia specific reporter line: *gfap:nls-mcherry*, which expresses nuclear-localized mCherry in telencephalic radial glia cells (Fig. S1B) under the control of the radial glia-specific *gfap* promotor (Bernardos and Raymond, 2006). Flow cytometry of dissociated forebrain cells from *gfap:nls-mcherry*; *elavl3:gfp* fish also identified a specific population of mCherry^low^/GFP^pos^ neurons (Fig. S1C,D). Quantification confirms similar percentages of mCherry^high^/GFP^neg^ and mCherry^low^/GFP^pos^ cells (Fig. S1E). To verify that the mCherry^low^/GFP^pos^ double-positive population is enriched with adult generated neurons, an EdU pulse-chase analysis was performed. Adult *her4.1:mcherry*; *elavl3*:*gfp* fish were injected three times with EdU in 12 h intervals to label sufficient numbers of proliferating cells in the adult brain. EdU incorporation was assessed at 2 h, 7 days or 4 weeks after the last injection to identify proliferating cells and their immediate progeny, early progeny and late progeny of the initially proliferating cells, respectively (Fig. 2A). After 2 h of chase, mCherry^high^/GFP^neg^ RG were the most intensively proliferating cell type as 7.8±2.1% of all cells were EdU^pos^, whereas only 0.8±0.3% of mCherry^low^/GFP^pos^ cells and 0.1±0.01% of mCherry^neg^/GFP^pos^ mature neurons were EdU^pos^ (Fig. 2B,C). In contrast, after 7 h of EdU chase, EdU incorporation in mCherry^low^/GFP^pos^ neurons was significantly increased to 11.6±2.1%, whereas it remained similar in mCherry^high^/GFP^neg^ RG (10.6±3.5%) and mCherry^neg^/GFP^pos^ neurons (1.5±0.8%). After 4 weeks of chase, EdU incorporation in mCherry^low^/GFP^pos^ neurons was reduced to 0.7±0.1% compared with the 7 days chase, mCherry^high^/GFP^neg^ RG showed a trend towards reduced EdU incorporation (3.9±1.6%), whereas mCherry^neg^/GFP^pos^ neurons showed no significant change (0.6±0.1%) (Fig. 2B,C). These data indicate that mCherry^low^/GFP^pos^ neurons are specifically enriched for adult-generated neurons and suggest that these cells represent a transient population that retains *her4.1*:mCherry at least for 7 days but for no more than 4 weeks after their generation from mCherry^high^/GFP^neg^ RG.

Together, these data provide substantial evidence that radial glia-derived NBNs in the adult zebrafish forebrain can be identified by retention of fluorescent proteins expressed in radial glia with simultaneous expression of a neuronal marker. Our analyses reveal a population of NBNs that is equal in number compared with the *her4.1*:mCherry^high^/*elavl3*:GFP^neg^ bona fide RG.

### Single cell sequencing identifies transcriptome profiles of radial glia, newborn neurons and mature neurons in the adult zebrafish telencephalon

**Fig. 3.**
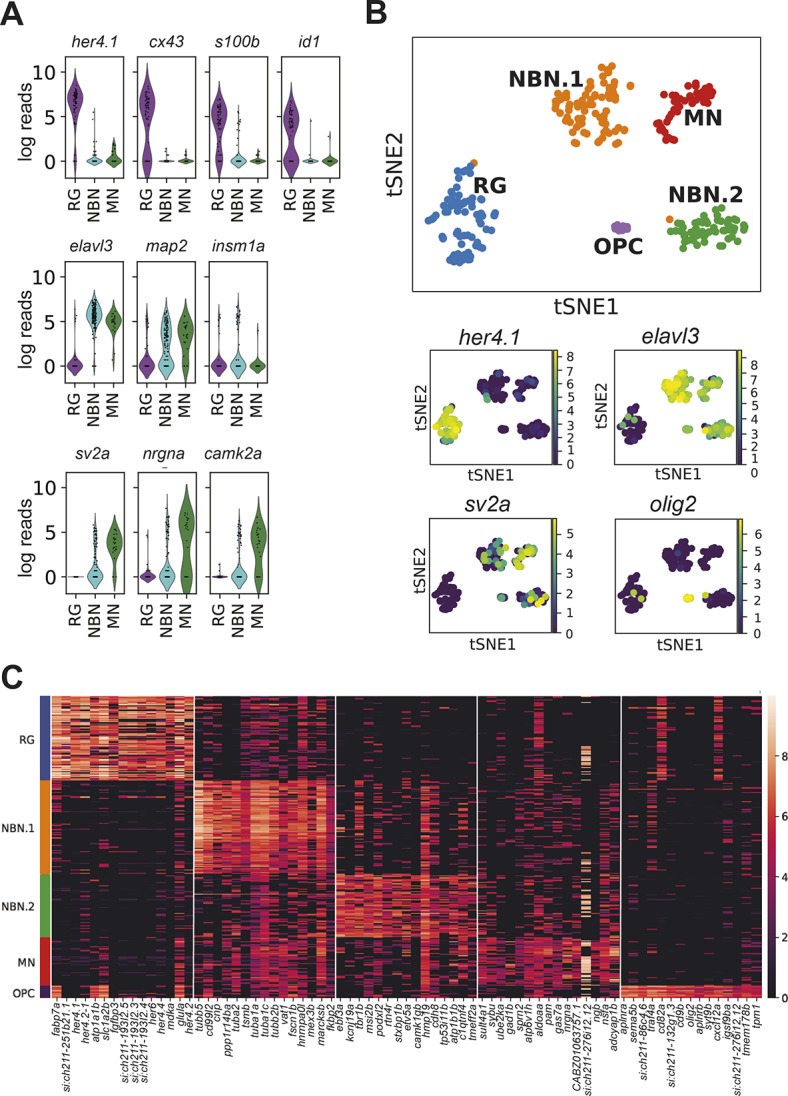
**Single cell RNA-seq reveals the diversity of radial glia progeny in the adult forebrain.** (A) Violin plots showing the expression of known radial glia markers (top), pan-neuronal markers (middle, *elavl3* and *map2*), an early neurogenic fate marker (middle, *insm1a*) and markers of mature synaptically integrated neurons (bottom). (B) tSNE plot revealing five different clusters from a total of 264 RG, NBN and MN cells (top). The cell number per cluster is: RG, 76 cells; NBN.1, 80 cells; NBN.2, 54 cells; MN, 44 cells; and OPC, 10 cells. Smaller panels underneath show expression of characteristic marker genes that separate the different clusters. (C) Heat map with the top 15 cluster-specific genes for the five identified clusters.

Next, using our lineage tracing protocol, we sorted 171 RG (mCherry^high^/GFP^neg^), 169 NBNs (mCherry^low^/GFP^pos^) and 30 mature neurons (MNs, mCherry^neg^/GFP^pos^) from the adult telencephalon of six fish and sequenced them using SMARTSeq2 (Picelli et al., 2014). On average, we detected 1408 expressed genes and 191,000 transcripts per cell. Of these, 264 cells (71%) passed our quality control: 76, 162 and 26 cells from the RG, NBN and MN FACS gates, respectively, were used for downstream analysis. Consistent with our lineage-tracing paradigm, cells that were sorted as RG, but not those sorted as neurons, expressed radial glia markers (*her4.1*, *cx43*, *id1* and *s100b*) (Grandel et al., 2006; Adolf et al., 2006; Kunze et al., 2009; Ganz et al., 2010; Rodriguez Viales et al., 2015) (Fig. 3A). Cells sorted as NBNs expressed pan-neuronal markers and early neuronal markers (*elavl3*, *map2* and *insm1a*) (Ferri and Levitt, 1993; Park et al., 2000; Raj et al., 2018), but showed minimal expression of mature synaptic proteins such as synaptic vesicle protein 2a (*sv2a*), neurogranin a (*nrgna*) and calmodulin-dependent kinase 2a (*camk2a*) (Zhong and Gerges, 2012), compared with mature neurons (Fig. 3A). We used Louvain clustering to identify five clusters with distinct transcriptomic profiles that we visualized by t-stochastic neighbor embedding (t-SNE) as well as principal component analysis (Fig. 3B,C; Fig. S2A,B). The cells from the three FACS gates segregated into five transcriptome clusters: one cluster (RG) consisted almost exclusively of cells sorted as RG, three clusters (NBN.1, NBN.2 and OPC) contained mostly cells sorted as NBN, and one mixed cluster (MN) contained cells from the NBN and MN gates (Fig. 3B; Fig. S2A). We assigned cell type identity to each cluster based on known marker genes (Fig. 3B): The radial glia marker *her4.1* was expressed specifically in the RG cluster, but not in the neuronal clusters NBN.1, NBN.2 and MN, which all expressed *elavl3*. *sv2a* was sparsely expressed in NBN.1 and NBN.2, but strongly enriched in cluster MN, confirming that MN contained mature neurons. Cells of the OPC cluster also expressed *elavl3* and were identified as oligodendrocyte progenitor cells by the expression of *olig2* (Park et al., 2007) (Fig. 3B,C). The transcriptome analyses identified several marker genes for each of the identified clusters, which allowed their biological characterization (Fig. 2C, Fig. S3, Table 1). The RG cluster expressed several known RG markers, e.g. *fabp7a* (also known as *blbp*), *her4.1* and *glula* (encoding glutamine synthase), but also novel markers such as *si:ch211-251b21.1* (encoding the glutamate receptor grik-1), *fgfbp3*, *atp1a1b*, *selenop* and *mdka*. The major pure NBN cluster (NBN.1) was characterized by specific expression of *tubb5*, *cd99l2*, *ppp1r14ba*, *cnp* and *tuba2*. The smaller pure NBN cluster (NBN.2) expressed the transcription factor *ebf3a*, *kcnj19a*, *tbr1b*, *msi2b* and *podxl2*. The mixed NBN and MN cluster (cluster MN) showed specific expression of *sult4a1*, *sybu*, *ube2ka*, *gad1b* and *sprn2*, while the OPC cluster predominantly expressed the marker genes *aplnra*, *sema5a*, *si:ch211-286c4.6* (encoding Cd59), *traf4a* and *cd82a*, which were recently identified as genes expressed in zebrafish OPCs (Kroehne et al., 2017; Raj et al., 2018). Together, these data show that radial glia-derived NBNs are a heterogeneous population, comprising at least three subsets (NBN.1, NBN.2 and a part of the MN cluster) with distinct transcriptional profiles. They are distinct from their radial glia precursors, but a subset of NBNs clusters together with mature, synaptically integrated neurons, suggesting that this population represents a more mature state of adult-generated neurons. Interestingly, one minor cluster, comprising NBNs, none of which expressed *her4.1*, but did express *elavl3*, was identified as oligodendrocyte precursors due to expression of cell-type specific markers (Fig. 3B,C; Fig. S4A). Retention of *mcherry*, expressed from a RG-specific promotor in these OPCs, as well as shared marker expression (e.g. *fabp7a*, *slc1a2b*, *atp1a1b*, *cd82a*, *cxcl12a* and *glula*; see Fig. 3C) supports their generation from RG in the adult zebrafish brain. In addition, flow cytometry of double transgenic fish that combine the RG-specific *her4.1*:mCherry reporter with the OPC-specific reporter *olig2*:*gfp* (Shin et al., 2003; Kroehne et al., 2017; Tsata et al., 2019) [Tg(*her4.1:mcherry*; *olig2:gfp*)] revealed that 28.6±3.9% of all *olig2*:GFP cells were mCherry^low^/GFP^high^ double positive (Fig. S4B,C). We also observed numerous cells showing high levels of mCherry fluorescence and low levels of *olig2*-driven GFP fluorescence, possibly representing nascent RG progeny differentiating towards OPCs (Fig. S4B). In summary, these data provide robust evidence that *her4.1*-expressing RG generate heterogeneous and diverse progeny comprising different neuronal and oligodendroglial cell types in the normal adult telencephalon.

**Table 1.**
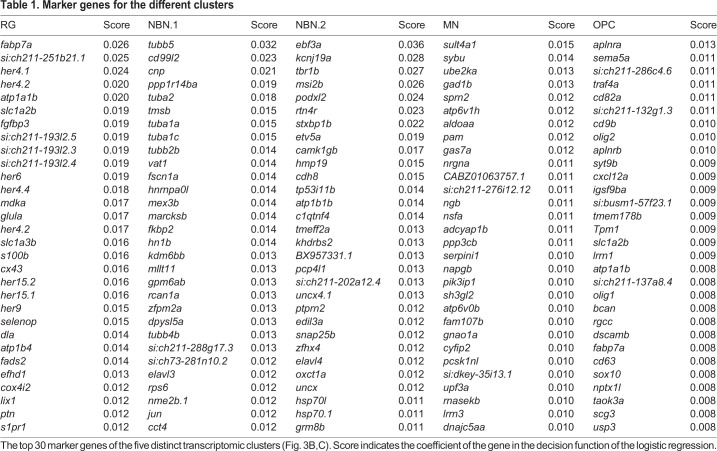
**Marker genes for the different clusters**

### Analysis of gene expression trajectories reveals dichotomic differentiation patterns of RG progeny

**Fig. 4.**
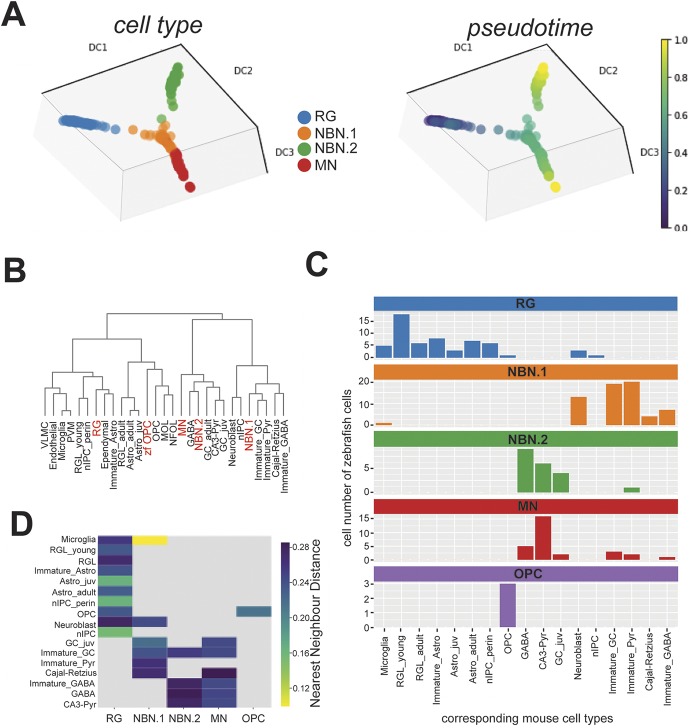
**The differentiation trajectory of zebrafish radial glia progeny clusters resembles mammalian adult neurogenesis.** (A) Lineage trajectory analysis of single cell RNAseq data from RG, NBNs and MNs of the adult zebrafish forebrain. Color coding corresponds to the different cell clusters identified in Fig. 2B (left) or indicates the pseudotime progression (right). (B) Unbiased hierarchical clustering of transcriptome profile from zebrafish cell clusters (red) with cell types from the developing and adult murine hippocampus (black), showing dispersed co-distribution of zebrafish cell clusters with related mammalian cell types. (C) Quantification of distribution of zebrafish cell clusters over the different corresponding murine cell types for RG (blue), NBN.1 cells (orange), NBN.2 cells (green), MNs (red) and OPCs (purple). Bars specify the number of zebrafish cells (*y*-axis) corresponding to the indicated murine cell type (*x*-axis). (D) Heatmap depicting the similarity of zebrafish cell clusters with their corresponding murine cell types based on the k-nearest neighbor distance. Lower distances indicate higher similarities.

To infer the differentiation trajectories of radial glia-derived NBNs, we performed pseudotime-based ordering of our single cell gene expression profiles based on diffusion maps (Fig. 4A). In contrast to dimensionality reduction by t-SNE, diffusion maps retain the global structure of the transcriptomes, such that continuous branching lineages can be visualized (Haghverdi et al., 2015). Using RG as a root group, pseudotemporal ordering using the diffusion pseudotime algorithm revealed the NBN.1 cluster as the differentiation state that is closely related to RG. From the NBN.1 cluster, a trajectory branchpoint was formed, leading to the MN cluster or the NBN.2 cluster, respectively. Among these two endpoints, the NBN.2 cluster was the most distant from RG (Fig. 4A). The OPC cluster was omitted from this analysis for clarity because it was transcriptionally disconnected from the other cells (Fig. S5A).

Next, we compared the different cell clusters with neurogenic cell types in the adult mammalian brain. A recent study generated sc-transcriptome profiles of radial glia, radial glia-like neural stem cells, their neuronal progeny and niche cells in the adult hippocampus and during hippocampal development (Hochgerner et al., 2018). For a combined cell type homology analysis, we transformed the transcriptomes of zebrafish and mouse cells into a transcriptome of all orthologues gene pairs (see Materials and Methods). Then, we performed hierarchical clustering of the zebrafish and murine cell types (Fig. 4B). The zebrafish cell clusters clustered together with different murine neuronal and glial cell types, indicating a dominance of cell type differences of the transcriptional profiles over species differences. In line with our assignment of cell identity, RG clustered together with murine ependymal cells and young astrocytes, and zebrafish OPCs clustered with murine OPCs and oligodendrocytes. Interestingly, NBN.1 cells formed a cluster with murine immature granule cells and pyramidal neurons, whereas NBN.2 and MN cells clustered together with mature neurons (Fig. 4B). All cell types showed a comparable distribution of similarity of zebrafish cells to their most similar murine counterpart, suggesting that, for each zebrafish cell type, corresponding cell populations exist in the mammalian hippocampus (Fig. S5C). To infer the heterogeneity of cell identity within the zebrafish cell clusters, we also determined the cell type of the single corresponding, most similar murine cell for each zebrafish cell. We omitted cells that show only limited similarity to mammalian cells, identified by Hochgerner et al. (2018) (see Fig. S5C and Materials and Methods). The results show that cells forming the RG cluster in the adult zebrafish brain mainly correspond to various glial cell types in the murine brain, i.e. astrocytes and radial glia-like neural stem cells (RGLs) of different maturation stages, neurogenic intermediate progenitor cells (nIPCs) with minor proportions corresponding to neuroblasts, OPCs and microglia. Zebrafish OPCs corresponded only to murine OPCs. Consistent with the pseudotime analysis, NBN.1 cells corresponded to neuroblasts and immature neurons of pyramidal, granule cell or GABAergic identity, while NBN.2 and MN cells mainly corresponded to mature neurons of GABAergic and pyramidal identity as well as juvenile granule cell neurons (Fig. 4C,D). In summary, these analyses reveal transcriptomic similarity of the neurogenic cell types of the adult zebrafish brain with those in the neurogenic niche of the developing and adult hippocampus. In particular, NBN.1 cells display pronounced similarity to neuroblasts and immature neurons, supporting their positioning as direct RG progeny in the pseudotime analysis.

**Fig. 5.**
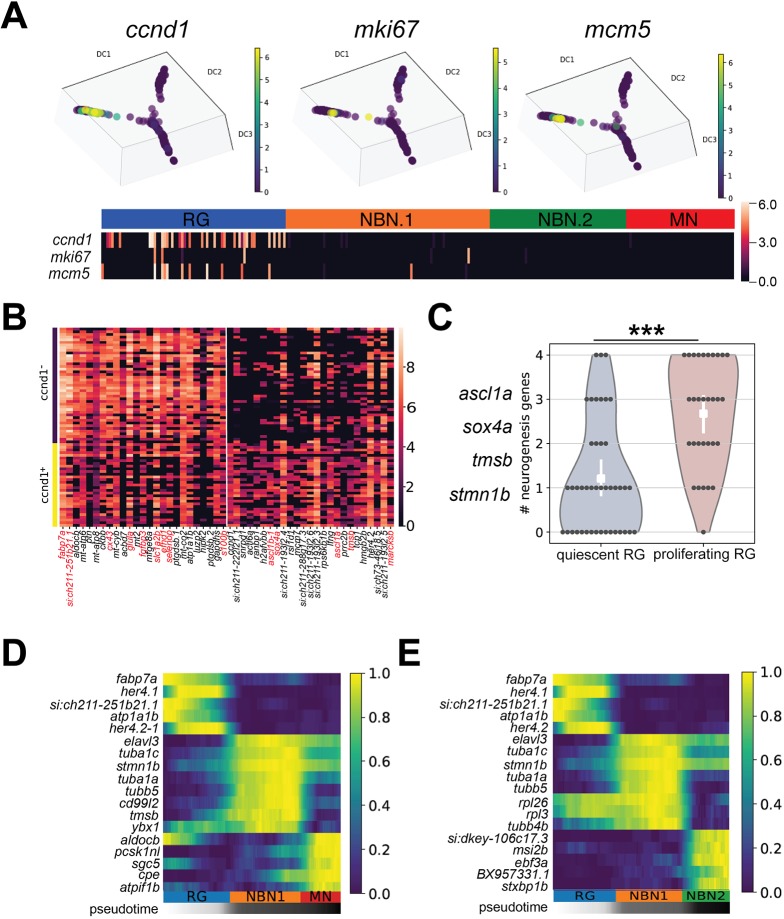
**Proliferation is confined to radial glia and is associated with neurogenic commitment**. (A) Expression pattern of the proliferation markers *ccnd1* (left), *mki67* (middle) and *mcm5* (right) along the trajectory path of RG differentiation. A heatmap indicating the co-expression of these genes in RG is shown at the bottom. (B) Heatmap showing differentially expressed genes in *ccnd1*^−^ quiescent RG and *ccnd1*^+^ proliferating RG. There is enrichment of classical RG markers in quiescent RG (left, red gene symbols), and of neurogenic fate determinants and NBN.1 marker genes in proliferating RG (right, red gene symbols). (C) Violin plot showing cumulative expression of the four most widely expressed neurogenic fate determinants and neuronal genes (left) in quiescent (gray) and proliferating (red) RG. Dots represent cells expressing the indicated number of genes. *n*=45 (quiescent) or 31 (proliferating). ***P<0.001 using a Mann-Whitney *U*-test. (D,E) Heatmaps showing gene expression dynamics of differentially expressed genes along the differentiation trajectory from RG to MNs (D) or from RG to NBN.2 cells (E). Genes (rows) are clustered and cells (columns) are ordered according to the pseudotime development. There is specific expression of *tubb5* in the NBN.1 and of *ebf3a* and *msi2b* in the NBN.2 cluster.

As a substantial proportion of NBN.1 cells showed similarity to neuroblast, which contain proliferating cells in mammalian adult neurogenic niches, we asked whether different subtypes of proliferating cells, e.g. radial glia, progenitors or neuroblasts (März et al., 2010a; Edelmann et al. 2013), are distributed along the pseudotime trajectory in zebrafish. Using *ccnd1*, *mcm5* and *mki67* as markers for proliferating cells, proliferation was confined to the RG cluster and no proliferating cells were found elsewhere within the RG lineage (Fig. 5A), consistent with the pronounced prevalence of EdU^+^ cells in RG at early EdU chase timepoints (see Fig. 2B,C). As *mcm5* and *mki67* expression is restricted to specific cell cycle phases (Ohtani et al., 1999; Sobecki et al., 2017), we used *ccnd1* as general proliferation marker to compare the transcriptome profile of *ccnd1^+^*, proliferating RG and *ccnd1*^−^ quiescent RG. Apart from the expected enrichment of proliferation genes, such as *snrpd1*, *actl6a* and *mcm7* (Hiraiwa et al., 1997; Batra et al., 2012; Krasteva et al., 2012), neuronal fate determinants, such as *ascl1a* and *sox4a*, and marker genes of the NBN.1 clusters, such as *tmsb* and *stmn1b*, were significantly enriched in proliferating RG (Figs 3C and 5B; Table 1; Table 2). In contrast, classical RG markers, such as *fabp7a*, *cx43*, *glula* (encoding glutamine synthase) and *s100b*, were enriched in quiescent RG (Figs 3C and 5B; Table 2). In support of a neurogenic commitment of proliferating RG, we found that 9% of the top 100 marker genes of NBN.1 cells were also enriched in proliferating RG, whereas only one of them was found to be enriched in quiescent RG (Fig. S5B). Strikingly, out of the four neuronal fate determinants/neuronal genes (*ascl1a*, *sox4a*, *tmsb* and *stmn1b*) that were most substantially expressed in RG (Table 2), 81% of proliferating RG expressed two or more of them, whereas only 27% of quiescent RG did (Fig. 5C). With the exception of *ascl1a*, which was virtually confined to RG, all neuronal genes were further strongly enriched in NBN.1 cells, suggesting that RG become committed to neurogenesis (indicated by consistent expression of neuronal fate determinants) and that many divisions from proliferating RG are neurogenic. Taken together, we identify RG as the only proliferating cell type in the cell trajectory derived from *her4.1*-positive RG and provide strong evidence that most proliferating RG are committed to neurogenesis.

**Table 2.**
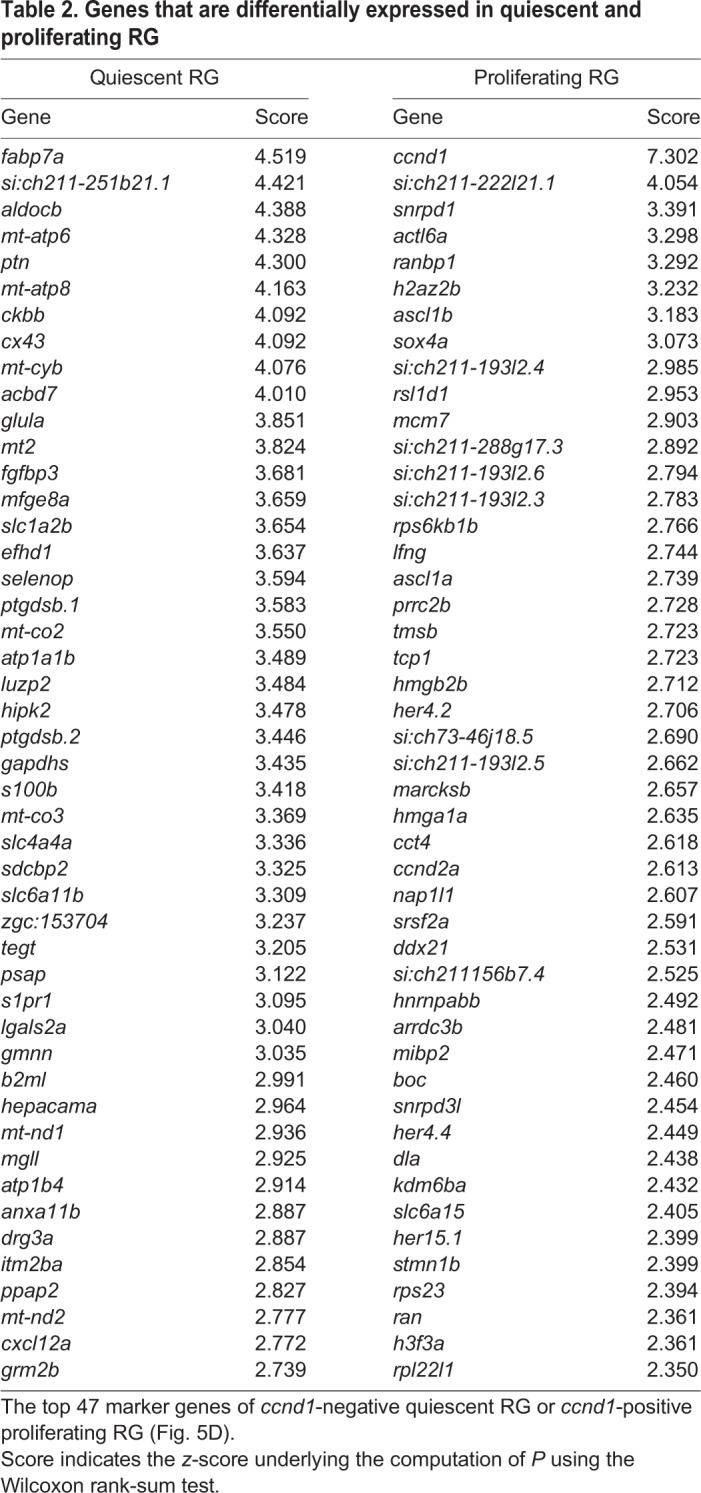
**Genes that** **are** **differentially expressed in quiescent and proliferating RG**

Next, we explored the gene expression changes upon the transition from RG to NBN.1 cells and towards MNs or NBN.2, respectively. Indeed, we observed waves of gene expression along the pseudotime ordering, identifying specific gene regulation along the differentiation trajectories. Consistent with the enrichment of the Notch target gene *her4.2* in proliferating RG (Fig. 5B), *her4.1* and *her 4.2* were increasingly expressed in RG that were advanced in the pseudotime trajectory (Fig. 5D,E). Furthermore, panneuronal markers such as *elavl3* were consistently upregulated in all neuronal cell types compared with RG, while *tubb5* and *tmsb*, or *ebf3a* and *msi2b* were specifically seen in NBN.1 or NBN.2 clusters, respectively (Fig. 5D,E).

In conclusion, pseudotemporal ordering of RG, NBNs and MNs identifies a differentiation trajectory from RG to NBN.1 cells, and reveals separate trajectories from NBN.1 towards NBN.2 cells or MNs, respectively. This conclusion is consistent with the comparison of zebrafish and murine neurogenic cell types, which reveals that RG, NBN.1 or NBN.2 and MN cells show transcriptomic similarities to cell types progressing in the known differentiation trajectory in this well-studied niche. Analysis of proliferation marker and neurogenic gene expression indicates that all analyzed proliferative cells in the her4.1-lineage are bona fide RG, and that a neuronal transcription program is prevalent in proliferating RG. Finally, differential gene expression analysis along the trajectory confirms specific marker genes for the NBN.1, the NBN.2 and the MN population.

### The NBN.1 and NBN.2 clusters form spatially defined populations of newborn neurons in the telencephalon and diencephalon, respectively

**Fig. 6.**
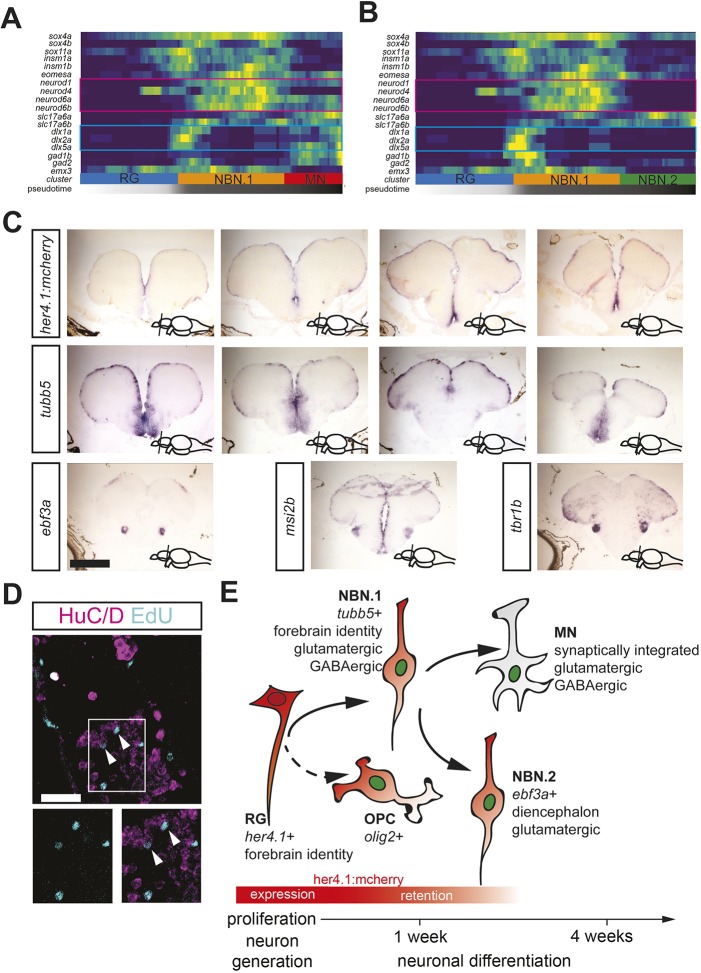
**The NBN clusters show diversity in cell fate determinant expression and anatomical localization in the adult forebrain.** (A,B) Heatmaps showing gene expression dynamics of proliferation markers, Notch targets, neurogenic fate determinants, telencephalic identity markers and neuronal subtype markers along the differentiation trajectory from RG to MNs (A) or from RG to NBN.2 cells (B). Genes (rows) are clustered and cells (columns) are ordered according to the pseudotime development. Magenta and turquoise frames mark identity markers for dorsal or ventral telencephalon, respectively. (C) *In situ* hybridization for *mcherry* in *her4.1*:*mcherry* reporter fish (top), the NBN.1 marker *tubb5* (middle), and the NBN.2 markers *ebf3a*, *msi2b* and *tbr1b* (bottom) in wild-type fish. There is consistent expression of NBN.2 markers in the lateral diencephalon. The rostrocaudal level of the section is indicated in the bottom right corner. (D) Optical section of immunostaining for the neuronal markers HuC/D (magenta) and EdU labeling (turquoise) in the vENT, indicating the localization of NBNs (arrowheads). (E) Schematic representation of the resulting model from this study. Scale bars: 200 µm in C; 20 µm in D.

To identify factors that mediate the separation of the NBN.2 and the MN cluster as the two more differentiated neuronal cell clusters, we investigated the expression of key cell fate meditators for pan-neurogenic fate (*sox4a*, *sox11a*, *insm1a*, *insm1b* and *eomesa*), dorsal telencephalon glutamatergic fate (*neurod4*, *neurod1*, *neurod6a* and *neurod6b*), ventral forebrain GABAergic fate (*dlx1a*, *dlx2a* and *dlx5a*) and glutamatergic or GABAergic neuron identity (*slc17a6a*, *slc17a6b*, *slc17a7a*, *gad1b* and *gad2*) along the differentiation trajectory. Pan-neurogenic markers and *eomesa* were induced in differentiated RG, were abundantly expressed in the NBN1 cluster, and continued to be expressed, although in fewer cells, in both the NBN.2 and the MN cluster (Fig. 6A,B). Similarly, the glutamatergic or GABAergic neuron markers were also expressed by NBN.1, NBN.2 and MN cells. In contrast, the markers of dorsal telencephalon glutamatergic identity (magenta frame in Fig. 6A) and ventral telencephalon GABAergic identity (turquoise frame in Fig. 6A) were expressed in the NBN.1 and the MN cluster, but absent in the NBN.2 cluster, suggesting that the NBN.2 population of newborn neurons does not have telencephalic identity.

Next, we determined the localization of NBN.1 and NBN.2 cells in the adult forebrain using *in situ* hybridization for the specific markers *tubb5* (NBN.1) or *ebf3a*, *msi2b* and *tbr1b* (all NBN.2) (Figs 3C and 5D,E). *tubb5* was expressed in the VZ and PVZ throughout the dorsal and ventral telencephalon, as well as in the anterior preoptic part of the diencephalon. Compared with the expression pattern of *mcherry* in the telencephalon of *her4.1:mcherry* fish, which is restricted to the VZ, the expression domain of *tubb5* was notably extended into the PVZ, which is a known location for newborn neurons (Grandel et al., 2006; Ganz et al., 2010; Furlan et al., 2017) (Fig. 6C). These results are consistent with the pseudotemporal positioning of *tubb5*-expressing NBN.1 cells as newborn neurons, which are most closely related to RG. In contrast, *ebf3a* expression was absent in the telencephalon, but was found specifically in the ventral entopeduncular nucleus (vENT) in the lateral anterior diencephalon (Mueller and Guo, 2009; Turner et al., 2016). *msi2b* and *tbr1b* also showed broader expression in the dorsal telencephalon, but consistently labeled the vENT (Fig. 6C), suggesting that the NBN.2 population consists of vENT NBNs. As adult neurogenesis was not reported in the vENT (Zupanc et al., 2005; Grandel et al., 2006), we investigated whether NBNs were located in the vENT. Adult fish were injected with EdU on three consecutive days and EdU^+^/HuC/D^+^ NBNs were analyzed 1 month after injection. As predicted, we found several EdU/HuC/D double-labeled neurons in the vENT (Fig. 6D), indicating that NBNs are added to this nucleus in adult stages. Thus, these results were consistent with our analysis of telencephalic marker expression (Fig. 6A) that suggested a location of NBN.2 cells outside of the telencephalon. Together, our spatial analysis shows that NBN.1 and NBN.2 cells are anatomically distinct populations of newborn neurons in the telencephalon and the diencephalon, respectively.

In conclusion, we have prospectively isolated RG, NBNs and MNs from the adult zebrafish forebrain, analyzed the kinetics of NBN generation and differentiation, and performed unbiased, single cell resolution transcriptome analysis of these cells. This analysis revealed distinct clusters of NBNs (Fig. 6E), which correspond to NBNs in the telencephalon and diencephalon. In addition, we identified specific marker genes for NBNs, which may facilitate further research on this important cell type in the adult vertebrate brain.

## DISCUSSION

The combination of lineage tracing and single cell sequencing is a powerful tool for dynamic analysis of somatic stem cell progeny and their role in tissue development, homeostasis and repair (Camp et al., 2018; Gerber et al., 2018; Kester and van Oudenaarden, 2018). Here, we have performed lineage tracing at the population level by using the inheritance of a fluorescent protein that is specifically expressed in RG by their progeny, similar to previous studies in the developing zebrafish telencephalon and regenerating adult retina (Fausett and Goldman, 2006; Furlan et al., 2017). We additionally used a fluorescent neuronal marker to identify NBNs derived from the RG lineage as cells that are double positive for both markers. An EdU pulse-chase experiment demonstrated that the double-positive cells are specifically enriched for adult-generated neurons and provides evidence that this population is transient and dynamic, because they are enriched for EdU-incorporating cells after a chase of 7 days, but not after 2 h or 4 weeks. The results suggest that the double positive cells lose their mCherry fluorescence between 7 days and 4 weeks after their generation by proliferating precursors (likely RG) and are replaced by newly generated cells at the latter timepoint, consistent with our lineage-tracing paradigm. The extent of RG proliferation measured by EdU incorporation is consistent with the frequency of proliferating RG in the literature (Dray et al., 2015). In addition, the persistence of her4.1-driven mCherry in the mCherry/GFP double-positive cells for up to 7 days is consistent with a previous *in vivo* imaging study, which reports inheritance of *gfap*:GFP in the differentiated daughter cells of RG for several days after division (Barbosa et al., 2015). Thus, these data verify our lineage-tracing approach and provide important insights into the time line and kinetics of neurogenesis and neuronal differentiation. Finally, we were able to enumerate for the first time the extent of radial glia-derived newborn neurons in the adult zebrafish forebrain, independent of thymidine analog administration. Using the *her4.1* promotor as a RG-specific driver, we find that circa 10% of sorted, live cells from the telencephalon are NBNs that still retain detectable levels of the RG-derived fluorescent marker. Our finding that *her4.1*:mCherry in the NBNs is detectable for several days after their generation from proliferating precursors (Fig. 2B,C) suggests that this NBN population is an accumulation of cells of different ages generated over 1 or 2 weeks, but over no more than 4 weeks. Consistently, our transcriptional analysis reveals a broad spectrum of differentiation states among NBNs, from NBN.1 cells that share substantial transcriptional overlap with RG (see Fig. 2C; Fig. S5A) to NBNs that form a common cluster with mature neurons (Fig. S2A). These results suggest that, with our lineage-tracing approach, we detect various differentiation states of NBNs, present at steady state in the uninjured telencephalon (Fig. 3B,C). The biological role of such a surprisingly high number of NBNs and the mechanisms of their integration are currently unknown, but our results pave the way for better investigation and manipulation of NBNs in the adult zebrafish brain. A recent report provided single cell transcriptional profiles of *her4.1*:GFP^pos^ radial glia and *her4.1*:GFP^neg^ neurons from the adult zebrafish telencephalon (Cosacak et al., 2019), but NBNs are likely under-represented in this analysis because *her4.1*:GFP^low^ cells were deliberately omitted from the analysis.

Using our lineage-tracing approach, we determined whether committed proliferating progenitors are present among the RG progeny in the adult forebrain. This analysis, at the population level, showed that RG are the only proliferating cell population within the sampled cells of the *her4.1*-lineage (Fig. 5A). Our estimation of the frequency of proliferating versus quiescent RG matches the values reported by Dray et al. (2015). We find that 11 out of 76 RG (14.5%) express the S/G2/M-phase-specific proliferation marker *mcm5* (Fig. 5A), corresponding to ∼15% of RG expressing the S/G2/M-marker PCNA or *mcm5*-driven EGFP at the protein level (Dray et al., 2015). The data are consistent with our comparison of zebrafish and murine neurogenic cells, where a subset of RG correspond to intermediate neurogenic progenitors, the major proliferating population in murine hippocampal adult neurogenesis. They also align with previous clonal analysis of RG progeny (Kroehne et al., 2011; Rothenaigner et al., 2011), which also argue against a major role of non-radial glia cells for amplification of RG progeny in zebrafish. Such non-glial precursors have been recently proposed as the quantitatively dominant proliferating cell type in the VZ/PVZ of the adult zebrafish telencephalon by live imaging *in vivo*. They were identified by the absence of destabilized RFP(dRFP) expressed under the control of the her4.1 promotor, and by the presence of *mcm5*-driven EGFP (Dray et al., 2015). As these progenitors are negative for classical glial markers, their lineage relationship with radial glia is currently unclear. Importantly, in our study, where mCherry is retained in RG progeny until 7 days after division (Fig. 2B,C), virtually all proliferating *her4.1*:mCherry^pos^ cells showed a transcriptome profile consistent with RG identity (Fig. 5A,B). Similarly, recent sc-RNAseq studies in the adult zebrafish brain with higher cell throughput, only identified cells with transcriptomic profiles of radial glia as proliferating neurogenic precursors (Cosacak et al., 2019; Yu and He, 2019). Our findings – that a subset of zebrafish RG corresponds to intermediate neurogenic progenitors, the major proliferating and amplifying population from the postnatal and adult murine hippocampus, while a NBN.1 subset corresponds to neuroblast, their direct progeny – support this view. Thus, our results argue against a direct lineage relationship between RG and these non-glial precursors, although we cannot exclude the possibility that the mCherry protein is diluted to undetectable levels in fast proliferating precursor progeny in radial glia, while it is still detectable in postmitotic neuronal progeny. We also find that gene expression in proliferating RG is linked to neurogenesis, as expression of neurogenic cell fate determinants (*ascl1a*, *ascl1b* and *sox4a*) and NBN.1 marker genes (*tmsb* and *marcksb*) is enriched in proliferating RG (Fig. 5B). These data are consistent with *in vivo* live-imaging analyses of RG proliferation that found most RG divisions generate differentiated, non-RG progeny (Barbosa et al., 2015; Dray et al., 2015), which were in part identified as HuC/D^pos^ neurons (Barbosa et al., 2015). Thus, our study complements the *in vivo* imaging of cellular behavior of neurogenic cell types in the adult zebrafish telencephalon with insights into the transcriptional regulation in quiescent and proliferating RG and their progeny at single cell resolution.

Another currently unresolved issue concerns the type of NBNs that are found in the adult zebrafish brain. In the telencephalon, GABAergic interneurons and aminergic neurons were previously found among NBNs (Adolf et al., 2006; Grandel et al., 2006), but the identity of a major fraction of NBNs was still unclear. Using unbiased genome-wide analysis of neuronal cell type markers, we found that the majority of newborn neurons expressed the glutamatergic markers *slc16a6a* or *slc16a6b*, while a smaller fraction expressed the GABAergic markers *gad1b* or *gad2*. In particular, in the NBN.1 cluster, but not in the NBN.2 cluster, pallial fate determinants of the *neurod* gene family were co-expressed with glutamatergic markers (Fig. 4A,B), indicating a pallial glutamatergic identity. Thus, this study shows for the first time that zebrafish constitutively generate pallial glutamatergic neurons at adult stage, raising the possibility that this neuronal class can also be efficiently regenerated after brain injury. Interestingly, we found a minor proportion of RG progeny that showed hallmarks of OPC identity, such as expression of *olig2*, *olig1*, *aplnrb* and *sox10* (Fig. 2B,C; Fig. S5A). OPCs with a very similar marker profile were also found in the juvenile zebrafish brain (Raj et al., 2018). Expression of marker genes for this population was also found prominently in sorted *olig2*:GFP^pos^ cells from the adult zebrafish spinal cord (Kroehne et al., 2017), strongly suggesting that these cells are bona fide OPCs. Moreover, 28.6% of *olig2*:GFP^pos^ cells showed low levels of *her4.1*:mCherry, while numerous cells showed intermediate levels of mCherry and GFP (Fig. S4B,C). Given that more than 25% of *olig2*:GFP^pos^ cells are also positive for *her4.1*:mCherry, the double-positive cells are far too numerous to be accounted for by the small subpopulation of olig2:GFP^pos^ precursors in the midline of the ventral telencephalon (März et al., 2010b). The persistence of *her4.1*-driven mCherry fluorescence in these cells suggests that they are generated by RG in the adult zebrafish brain, in addition to the self-renewal capabilities of OPCs. Thus, we provide the first evidence here for the possible generation of OPCs from RG in the adult zebrafish brain. Whether specialized subsets of RG for generation of neurons and OPCs exists is currently unclear, but future scRNASeq studies in the zebrafish brain with higher cell throughput will enable better clarification of this issue.

Moreover, in addition to the NBN.1 population, which represents the nascent RG progeny, according to analysis by pseudotime ordering, transcriptional overlap and comparison with corresponding mammalian cell types (Fig. 4A,C,D; Fig. S5A), we also identify two more mature populations of adult-generated neurons. One of these populations forms a common cluster with mature neurons that do not retain her4.1-driven mCherry fluorescence and expresses telencephalic identity markers such as *neurod* and *dlx* genes. The NBNs in this cluster are considered to be generated from adult RG within the last 4 weeks, based on the retention of *her4.1*:mCherry (Fig. 2B,C), but are advanced in their maturation as many of them already express maturity markers such as *sv2a* (compare Fig. 3B and Fig. S2A). Within the MN cluster, cells expressing glutamatergic or GABAergic markers are found. Likely, the small number of analyzed cells in this cluster precludes the separation of these cell types into separate clusters, as found recently (Cosacak et al., 2019). The second, matured NBN.2 population lacks telencephalic markers and consists mostly of glutamatergic neurons (Fig. 6B). Spatial analysis of marker genes identifies these NBNs as belonging to the vENT in the diencephalon, which is included in our analysis, because the anterior diencephalon cannot be surgically separated from the telencephalon at the whole-mount level. Together with our observation that EdU^pos^ NBNs are added to this nucleus in adult stages, this argues that these NBNs are likely derived from diencephalic RG; further direct lineage tracing of diencephalic RG will be necessary to test this hypothesis.

Overall, we find that transcriptome analysis at single cell resolution can distinguish functionally and spatially defined subpopulations of RG progeny in the adult zebrafish brain. The insights presented here also highlight the heterogeneity of RG reporter-positive cells in the adult zebrafish brain, and advocate the use of multiple reporters to clearly define cell types.

Importantly, we identify here marker genes for functional subpopulations of NBNs, such as *tubb5* and *ebf3a* for the NBN.1 and NBN.2 population, respectively. Another recent study also suggested a population of *tubb5*- and *neurod*-expressing cells as nascent, telencephalic neurons in the juvenile zebrafish brain (Raj et al., 2018). Our results are consistent with this conclusion, based on also using lineage-tracing, pseudotime-ordering and location analysis in the adult brain. Identification of specific markers for NBNs – which are currently elusive – will greatly facilitate the study of their biology, because it enables the generation of transgenic tools for studying these cells, e.g. through CRISPR-Cas9-mediated generation of reporters, *Cre*ERT2 lines (Auer et al., 2014; Kesavan et al., 2017, 2018) or effector lines (NTR, dCas9-effectors, etc.).

## MATERIALS AND METHODS

### Zebrafish

Zebrafish were bred and maintained according to standard procedures. All animal procedures were approved by the Regierungspräsidium Dresden (permit AZ 24-9165.40-1/2007, 24-9165.40-1/2013-1, TVV 44/2017). Wild-type experimental fish were in the AB background. Fish were raised at a density of 50-60 fish/12 l tank (Brand et al., 2002). Fish of either sex were used.

### Transgenic fish lines

The *her4.1*:*mcherry*, the *elavl3*:*gfp* and the *olig2*:*gfp* lines have been described previously (Kroehne et al., 2011; Park et al., 2000; Shin et al., 2003). For the generation of the *gfap* reporter line *Tg(gfap:nls-mcherry)*, the *gfap* promoter (Bernardos and Raymond, 2006) was PCR amplified (gfap-for, 5′-gggccCACCTTTGGGATGTAGTGGAACGGG; gfap-rev, 5′-ggccggccAGGAACGCTGGGACTCCATGGTGGA) flanked by ApaI and FseI restriction sites that allowed substitution of the *her4.1* promoter of the *her4.1:mCherry-T2A-CreER^T2^* plasmid (Kroehne et al., 2011). The lowercase letters indicate the non-sequence-specific part of the primers that harbours the restriction sites (ApaI in the forward primer and FseI in the reverse primer). Correspondingly, the uppercase letters indicate the part of the primer that corresponds to the sequence of the *gfap* promotor. The mCherry reporter was PCR amplified and flanked by unique restriction sites using the primers Cherry-Fse-for (atatGGCCGGCCgccaccatggctccaaagaagaagcgtaaggtaatggccatcatcaaggagttcatc) and Cherry-Asc-rev (cgccGGCGCGCCgaattaaaaaacctcccacacc). The nuclear localization sequence (nls) was introduced into the sequence by a 5′ overhang of the PCR forward primer. The PCR product was subloned into the pCR2.1-TOPO vector. Next, the TOPO vector with the reporter and the *pTol(gfap:mcherry-T2A-CreERT2)* construct were digested using the enzymes AscI and FseI, and ligated to replace the mCherry-CreERT2 cassette with the nls-reporter. For germ-line transformation, linearized plasmid DNA with transposase mRNA was injected into fertilized eggs (F0), raised to adulthood and crossed to AB wild-type fish as previously described (Kawakami et al., 2004). For the identification of transgenic fish, F1 embryos were examined under a fluorescent microscope and positive embryos were raised.

### Tissue dissociation and cell sorting

Wild type or *her4.1*:*mcherry*;*elavl3*:*GFP* transgenic fish were sacrificed by an overdose (0.024%) of MS-222 (Sigma) (Brand et al., 2002) until cessation of opercular movement and transferred to ice-cold HBSS. The dorsal skull plates were removed using forceps, incisions were made caudal to the olfactory bulbs and rostral to the midbrain, and the intermittend telencephalon and anterior diencephalon was transferred to Eppendorf tubes. Of note, this dissection protocol will yield cells from both the telencephalon and the anterior diencephalon, as no clear anatomical separation exists between these two parts at the level of whole-mount brain tissue. Single cell suspensions were prepared from single telencephali/anterior diencephali using the Neural Tissue Dissociation Kit (Miltenyi Bioscience) with enzymatic digestion at 28°C instead of 37°C to prevent heat stress of the zebrafish cells. Prior to sorting, all samples were passed through a 20 μm cell strainer to remove cell aggregates and Calcein Blue-AM (Thermo Bioscience) was added to a final concentration of 10 µM.

Cells expressing mCherry and GFP were detected on a LSR II cell sorter (BD Biosciences) after 488 nm excitation and a bandpass-filter 564/42 nm and 530/30 nm, respectively. Calcein Blue was detected after 405 nm excitation and a bandpass-filter 450/40 nm. Forward and side scatter were used to select all events that show a characteristic scatter profile of zebrafish brain cells. From this selection, all events that showed incorporation of the Calcein Blue were gated as living cells. Living cells were plotted for GFP and mCherry fluorescence to gate for the respective cell populations. Detection of all fluorescent samples was controlled against unstained wild-type cells. Single cells gated as RG (mCherry^high^/GFP^neg^), NBN (mCherry^low^/GFP^pos^) and MN (mCherry^neg^/GFP^pos^) were sorted into single cavities of 96-well plates containing 2 µl lysis buffer using index sort mode and a 100 µm nozzle. The samples were immediately frozen until preparation of sequencing libraries.

### Analysis of EdU incorporation in fluorescent cell populations

For the analysis of EdU incorporation into the different cell types of forebrains from *her4.1*:*mcherry*;*elavl3*:*GFP* transgenic fish, 7-10 forebrains were pooled for one sample. At different times after EdU injection, forebrains were dissected, dissociated and processed for FACS as described above. Single cells gated as mCherry^high^/GFP^neg^, mCherry^low^/GFP^pos^ and mCherry^neg^/GFP^pos^ were sorted into Eppendorf tubes containing 200 µl Click-IT fixative (Thermo) using a 100 µm nozzle. For the two mCherry^pos^ populations, 8000-30,000 cells were sorted; for the mCherry^neg^/GFP^pos^ population, sorting was stopped at 100,000 cells. Cells were incubated for 15 min in the dark and EdU was detected using the Click-IT Plus EdU AlexaFluor 647 Flow Cytometry Assay Kit (Thermo) according to the manufacturer's instructions. After EdU detection, the cell pellet was resuspended in 200 µl HBSS (Thermo), including 10 µg/ml (w/v) and incubated for 10 min at room temperature to stain the DNA in cell nuclei. Cells that are positive for AlexaFluor 647-labeled EdU and/or Hoechst were detected on a LSR II cell sorter (BD Biosciences) using 633 nm and 405 nm excitation, and a bandpass filter (660/20 nm and 530/30 nm, respectively).

### Imaging flow cytometry

For imaging flow cytometry, dissected zebrafish forebrains were dissociated and processed as for conventional flow cytometry (see above). The dissociated cells were analyzed for reporter fluorescence (GFP and mCherry) and Calcein Blue as viability marker using an ImageStream X Mark II imaging flow cytometer (Amnis), after gating forebrain cells for singlets (aspect ratio versus area). Data were processed offline using IDEAS software (Amnis). At least 20 individual images of double-positive (GFP^+^/mCherry^+^) cells, mCherry only-positive cells (GFP^−^/mCherry^+^), GFP only-positive cells (GFP^+^/mCherry^−^) and negative cells (GFP^−^/mCherry^−^) were analyzed and quantified for the appearance of GFP and DsRED within the same cell, doublets and cells with attached fluorescent debris.

### RNA sequencing

Cells were sorted by FACS into a 96-well plate containing 2 µl of nuclease free water with 0.2% Triton-X 100 and 4 U murine RNase Inhibitor (NEB), spun down and frozen at −80°C. After thawing the samples, 2 µl of a primer mix was added [5 mM dNTP (Invitrogen), 0.5 µM dT-primer* and 4 U RNase Inhibitor (NEB)]. RNA was denatured for 3 min at 72°C and the reverse transcription was performed at 42°C for 90 min after filling up to 10 µl with reverse transcription buffer mix for a final concentration of 1× superscript II buffer (Invitrogen), 1 M betaine, 5 mM DTT, 6 mM MgCl_2_, 1 µM TSO-primer*, 9 U RNase Inhibitor and 90 U Superscript II. After synthesis, the reverse transcriptase was inactivated at 70°C for 15 min. The cDNA was amplified using Kapa HiFi HotStart Readymix (Peqlab) at a final 1× concentration and 0.1 µM UP-primer under the following cycling conditions: initial denaturation at 98°C for 3 min; 22 cycles of 98°C for 20 s, 67°C for 15 s and 72°C for 6 min; and final elongation at 72°C for 5 min. The amplified cDNA was purified using 1× volume of hydrophobic Sera-Mag SpeedBeads (GE Healthcare) and DNA is eluted in 12 µl nuclease free water. The concentration of the samples was measured with a Tecan plate reader Infinite 200 pro in 384 well black flat bottom low-volume plates (Corning) using AccuBlue Broad range chemistry (Biotium).

For library preparation, 700 pg cDNA in 2 µl were mixed with 0.5 µl Tagment DNA Enzyme and 2.5 µl Tagment DNA Buffer (Nextera, Illumina) and tagmented at 55°C for 5 min. Subsequently, Illumina indices were added during PCR (72°C for 3 min; 98°C for 30 s; 12 cycles of 98°C for 10 s, 63°C for 20 s and 72°C for 1 min; and 72°C for 5 min) with 1× concentrated KAPA Hifi HotStart Ready Mix and 0.7 µM dual indexing primers. After PCR, libraries were quantified with AccuBlue Broad range chemistry, equimolarly pooled and purified twice with 1× volume Sera-Mag SpeedBeads. This was followed by Illumina sequencing on a Nextseq500 aiming at an average sequencing depth of 0.5 million reads per cell. Primers used were as follows: dT-primer, C6-aminolinker-AAGCAGTGGTATCAACGCAGAGTCGACTTTTTTTTTTTTTTTTTTTTTTTTTTTTTTVN, where N represents a random base and V any base beside thymidine; TSO-primer, AAGCAGTGGTATCAACGCAGAGTACATrGrGrG, where rG stands for ribo-guanosine; UP-primer, AAGCAGTGGTATCAACGCAGAGT.

### Computational analysis

Libraries were sequenced on an Illumina NextSeq 500 system, resulting in about 250,000 to 900,000 single end reads per cell. FastQC was used to examine quality of the reads after sequencing. Alignment of the reads to the GRCz10 reference, inclusive the 92 ERCC Spike-In transcripts, was carried out with GSNAP (v 2017-08-15), and Ensembl gene annotation version 87 was used to detect exon spanning reads. featureCounts (v1.5.3) was used with the same Ensembl annotation to count the uniquely aligned reads to the genes and to create a counts table.

To identify low quality cells, quality control metrics were calculated from the raw count matrix in R using the package scater (McCarthy et al., 2017). Only cells that had more 4.5 log10-transformed total reads, between 2.7 and 3.9 log10-transformed features with more than one read, less than 50% of the reads in the top 50 highest expressed genes, less than 20% reads from mitochondrial genes, less than 25% reads from spike-ins and for which more than 20% of the reads were mapped to the genome were used for further analysis. Using these criteria, from the 370 cells that were sequenced, 264 cells (71%) were used for further analysis. Genes that were detected in fewer than three cells were not considered for downstream analysis.

Further analysis was performed using the scanpy package (master branch commit 623f0d4) (Wolf et al., 2018, 2019). Using the scanpy function highly_variable_genes with max_mean set to 8.0, 5142 highly variable genes were identified from the log-transformed raw counts. Read counts were normalized using the deconvolution method implemented in the scran package (Lun et al., 2016 preprint), log-transformed, and scaled to unit variance and zero mean. A principal component analysis was performed on the highly variable genes. t-SNE dimensionality reduction was performed using the first 10 principal components (Ulyanov, 2018; Amir et al., 2013; Maaten and Hinton 2008). Based on the first 10 principal components, the diffusion map was calculated using a Gaussian kernel and sigma was implicitly determined by the distance to the five nearest neighbors (Haghverdi et al., 2015, Buettner and Theis 2012; Coifman et al., 2005). For the clustering, the 10 nearest neighbor graph was computed based on the first 10 principal components (McInnes and Healy, 2018 preprint). The neighborhood graph was used to perform Louvain clustering with the resolution parameter set to 0.5 (Blondel et al., 2008; Levine et al., 2015; Traag, 2015). Marker genes were detected using logistic regression on the raw count matrix as implemented in scanpy. The identification of cell identities in these clusters was based on examining these marker genes. For further analysis, the OPCs were dropped and a principal component analysis was performed on the resulting count matrix. A diffusion map was calculated with the same parameters as above. Diffusion pseudotime was computed with the dpt function of scanpy using the randomly chosen RG cell ‘RG-5_03_D09’ as root (Haghverdi et al., 2015; Wolf et al., 2018b preprint). To identify genes that mark the transitions between clusters, a pairwise differential expression analysis using a Wilcoxon rank-sum test was performed. For visualization of gene expression along diffusion pseudotime, a running average with a window width of 15 cells was computed.

Proliferative RG were defined as those that had one or more reads of ccnd1. All other RG were classified as non-proliferative. Differential expression analysis between the two groups was performed using a Wilcoxon rank-sum test. To examine the statistical significance of the over-representation of the top 100 marker genes for the NBN.1 cluster in the top 100 genes that are higher expressed in proliferative RG compared with quiescent RG a hypergeometric test was performed. From the 11 genes that were present in both, the top 100 marker genes for the NBN.1 cluster and the top 100 genes that are higher expressed in proliferative RG, four genes associated with neurogenesis were curated. For each cell, the number of expressed genes (>0 reads) among those four genes was calculated. To test the statistical significance of the increase in the number of cells that express genes associated with neurogenesis in proliferative RG, a one-sided Wilcoxon rank-sum test was performed.

### Comparison with murine cell types

For the combined cell type homology analysis, scanpy version 1.4.4 was used. Read counts and metadata on cell type identities from the developing mouse dentate gyrus were acquired from GEO accession number GSE104323. Read counts from zebrafish and mouse were normalized using the scanpy function normalize_per_gene and log-transformed using the log1p function of scanpy. Next, the normalized count matrices for zebrafish and mouse were transformed into a count matrix for all orthologues gene pairs in the following way. A list of all pairs of orthologues genes between mouse (GRCm38.p6) and zebrafish (GRCz11) was downloaded from www.ensembl.org/biomart/martview/ (database, Ensembl Genes 97; Dataset, zebrafish genes (GRCz11); filters, Orthologous Mouse Genes: Only; Attributes, Gene stable ID, Gene name, Mouse gene stable ID, Mouse gene name). It should be noted, that this is not a list of only one-to-one matches, i.e. a single species-specific gene can be contained in multiple pairs of orthologues genes. A cross-species count matrix was constructed in which each row corresponds to an orthologues gene pair and the columns correspond to the cells from both species. Let *z*^*j*^_*a*_ denote the normalized expression of zebrafish gene *a* in zebrafish cell *j* and *m*^*k*^_*b*_ denote the normalized expression of mouse gene *b* in mouse cell *k*. Then, the elements of this cross-species count matrix were assigned as
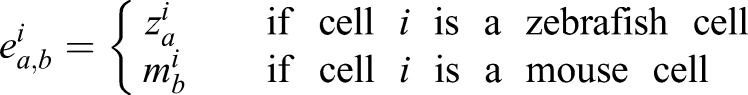
for all mouse and zebrafish cells *i*, and all orthologues gene pairs *a*, *b*. To align mouse and zebrafish data, batch correction was performed using the combat function of scanpy with the species as the batch key. Highly variable orthologous gene pairs were selected using the scanpy function highly_variable_genes with parameter flavor ‘cell_ranger’. A principal component analysis was performed on these highly variable orthologues gene pairs. Hierarchical clustering in the space of the first 50 principal components using a Pearson correlation distance was performed using the dendrogram function in scanpy. For each zebrafish cell, the Pearson correlation distance to and the cell type of the nearest mouse cell in the space of the first 50 principal components were computed. To reduce the number of false positives, zebrafish cells with distances to mouse cells above the 66% quantile of the distance distribution were disregarded from the analysis.

### Tissue preparation

Brains were exposed *in situ* and fixed at 4°C overnight in 2-4% paraformaldehyde/0.1 M phosphate buffer (pH 7.5). They were washed twice with phosphate-buffered saline (PBS) and transferred for decalcification and cryoprotection to 20% sucrose/20% EDTA in 0.1 M phosphate buffer (pH 7.5). Brains were frozen in 7.5% gelatine/20% sucrose and cut at 14 µm. Sections were stored at −20°C.

### *In situ* hybridization

RNA *in situ* hybridization on sections and on whole-mount brains and RNA probe generation was essentially performed as previously described (Ganz et al., 2012). Briefly, after defrosting at room temperature, sections were rehydrated for 15 min in PBS with 0.3% TritonX (PBSTx) and incubated with the probe overnight at 62-65°C. Information on the antisense *in situ* riboprobe for *tbr1b* can be found elsewhere (Ganz et al., 2012). The *in situ* probe for *tubb5* was obtained from the Zebrafish Gene Collection (cDNA clone MGC:85895). Probes for ebf3a and msi2b were cloned from zebrafish embryonic cDNA with the following primers: *ebf3a*-F, CAGCCAGTGGAGATCGAAAGGACAG; *ebf3a*-R, TGCCGTAGGGAGAGTTCGCAGAGGA; *msi2b*-F, GTTAGCCATGGAGGGAGACG; *msi2b*-R, GCGTCTTGGAAAGGCAACTT). The sections were washed at 60-65°C in washing solution (1×SSC, 50% deionized formamide) for 1×15 min and 2×30 min followed by 2×30 min MAB with 0.1% Tween-20 (MABT) washes. Sections were incubated for 1 h at room temperature in 2% DIG-blocking reagent (Roche) and incubated with anti-DIG antibody (Roche Diagnostics, sheep, polyclonal, Fab fragments conjugated to alkaline phosphatase, 11093274910) diluted 1:4000 in 2% DIG-blocking reagent overnight at 4°C. Subsequently, sections were washed for 4×20 min in MABT, equilibrated with staining buffer and stained with the substrate NBT/BCIP. The staining was controlled using a stereomicroscope. Finally, sections were washed for 2×5 min in PBS, postfixed with 4% PFA for 20-30 min, washed again for 2×10 min in PBS and mounted with 70% glycerol in PBS. All washing steps were performed on a shaker, all incubation steps in a humid chamber.

### Immunohistochemistry and EdU detection

Immunohistochemistry was carried out as previously described (Kroehne et al., 2011). Briefly, for detection of HuC/D, sections were subjected to antigen retrieval by 15 min incubation in 10 mM citrate buffer (pH 6.8). Primary and secondary antibodies were incubated in PBS with 0.3% Triton X-100 (PBS TX). Tissue sections were incubated with primary antibodies overnight at 4°C and secondary antibodies for 1 h at room temperature. The slides were then washed in PBS TX and mounted. We used primary antibodies to HuC/D (Elavl3) (mouse, Thermo Fisher Scientific, A21271, 1/250). For primary antibody validation, see www.thermofisher.com/antibody/product/HuC-HuD-Antibody-clone-16A11-Monoclonal/A-21271. Alexa Fluor 633-conjugated secondary antibodies were used (Thermo Fisher Scientific, A-21050, A21422; 1/750). EdU was detected with the EdU Alexafluor 488 Plus Imaging Kit (Thermo Fisher Scientific, C10337) according to the manufacturer's instructions.

### EdU injection

Adult (6-12 months old) fish were anaesthetized with 0.01% MS-222 (Sigma) in fish water (Brand et al., 2002) until sedation and injected intraperitoneally with 20 µl of 5 mg/ml EdU in PBS either for three times with 12 h interinjection interval (for analysis of EdU incorporation in *her4.1:mcherry*; *elavl3*:*gfp*-double transgenic fish) or for three times on consecutive days (for detection of adult-generated neurons in the diencephalon). After further survival for up to 1 month, fish were sacrificed by overdose with 0.024% MS-222 (Sigma) in fish water (Brand et al., 2002) until cessation of opercular movement. Subsequently, brains were dissected and processed for FACS (for analysis of EdU incorporation in *her4.1:mcherry*; *elavl3*:*gfp*-double transgenic fish) or processed for cryosectioning (detection of adult-generated neurons in the diencephalon).

### Image acquisition and processing

Images of brain sections, stained using *in situ* hybridization or immunofluorescence were imaged with a Zeiss Axioimager Z1 microscope with Apotome structured illumination to achieve optical sections in fluorescent images, using a Zeiss Plan-Apochromat 5×/0.16 and Zeiss Plan-Apochromat 40×0.95 objectives. To minimize crosstalk between the channels in multicolored specimens, sequential image acquisition was performed. The images were processed using ImageJ v.1.44 (rsb.info.nih.gov/ij/) and Adobe Photoshop CC 2015. Figures were assembled using Adobe Illustrator CC 2015.

### Statistics

For statistical analysis, *n* indicates the number of forebrain cell pools, where cells from 7-10 telencephali were pooled (EdU incorporation) or the number of single quiescent od proliferating RG cells (neurogenic commitment in proliferating RG). Statistical significance was determined using one way ANOVA with Welch's correction for heteroskedatic data followed by Bonferroni's multiple comparison *post hoc* test (EdU incorporation) or using a one-sided Mann and Whitney *U*-test (neurogenic commitment in proliferating RG). Statistical tests were performed with Graph Pad Prism 8. No animals or data points were excluded from the analyses. Cells in the single cell analysis were excluded based on pre-determined technical quality control parameters. No methods were used to pre-determine sample size or randomize group assignment.

## Supplementary Material

Supplementary information

## References

[DEV185595C1] Adolf B., Chapouton P., Lam C. S., Topp S., Tannhäuser B., Strähle U., Götz M. and Bally-Cuif L. (2006). Conserved and acquired features of adult neurogenesis in the zebrafish telencephalon. *Dev. Biol.* 295, 278-293. 10.1016/j.ydbio.2006.03.023 16828638

[DEV185595C2] Aimone J. B., Li Y., Lee S. W., Clemenson G. D., Deng W. and Gage F. H. (2014). Regulation and function of adult neurogenesis: from genes to cognition. *Physiol. Rev.* 94, 991-1026. 10.1152/physrev.00004.2014 25287858 PMC4280160

[DEV185595C3] Alemany A., Florescu M., Baron C. S., Peterson-Maduro J. and van Oudenaarden A. (2018). Whole-organism clone tracing using single-cell sequencing. *Nature* 556, 108-112. 10.1038/nature25969 29590089

[DEV185595C4] Alunni A. and Bally-Cuif L. (2016). A comparative view of regenerative neurogenesis in vertebrates. *Development* 143, 741-753. 10.1242/dev.122796 26932669 PMC4813331

[DEV185595C5] Alunni A., Hermel J.-M., Heuze A., Bourrat F., Jamen F. and Joly J. S. (2010). Evidence for neural stem cells in the medaka optic tectum proliferation zones. *Dev. Neurobiol.* 70, 693-713. 10.1002/dneu.20799 20506557

[DEV185595C6] Amamoto R., Huerta V. G., Takahashi E., Dai G., Grant A. K., Fu Z. and Arlotta P. (2016). Adult axolotls can regenerate original neuronal diversity in response to brain injury. *Elife* 5, e13998 10.7554/eLife.13998.025 27156560 PMC4861602

[DEV185595C7] Amir E.-A. D., Davis K. L., Tadmor M. D., Simonds E. F., Levine J. H., Bendall S. C., Shenfeld D. K., Krishnaswamy S., Nolan G. P. and Pe'er D. (2013). viSNE enables visualization of high dimensional single-cell data and reveals phenotypic heterogeneity of leukemia. *Nat. Biotechnol.* 31, 545-552. 10.1038/nbt.2594 23685480 PMC4076922

[DEV185595C8] Auer T. O., Duroure K., De Cian A., Concordet J.-P. and Del Bene F. (2014). Highly efficient CRISPR/Cas9-mediated knock-in in zebrafish by homology- independent DNA repair. *Genome Res.* 24, 142-153. 10.1101/gr.161638.113 24179142 PMC3875856

[DEV185595C9] Barbosa J. S., Sanchez-Gonzalez R., Di Giaimo R., Baumgart E. V., Theis F. J., Götz M. and Ninkovic J. (2015). Neurodevelopment. Live imaging of adult neural stem cell behavior in the intact and injured zebrafish brain. *Science* 348, 789-793. 10.1126/science.aaa2729 25977550

[DEV185595C10] Batra R., Harder N., Gogolin S., Diessl N., Soons Z., Jäger-Schmidt C., Lawerenz C., Eils R., Rohr K., Westermann F. et al. (2012). Time-lapse imaging of neuroblastoma cells to determine cell fate upon gene knockdown. *PLoS ONE* 7, e50988 10.1371/journal.pone.0050988 23251412 PMC3521006

[DEV185595C11] Baumgart E. V., Barbosa J. S., Bally-Cuif L., Götz M. and Ninkovic J. (2012). Stab wound injury of the zebrafish telencephalon: a model for comparative analysis of reactive gliosis. *Glia* 60, 343-357. 10.1002/glia.22269 22105794

[DEV185595C12] Berg D. A., Kirkham M., Beljajeva A., Knapp D., Habermann B., Ryge J., Tanaka E. M. and Simon A. (2010). Efficient regeneration by activation of neurogenesis in homeostatically quiescent regions of the adult vertebrate brain. *Development* 137, 4127-4134. 10.1242/dev.055541 21068061

[DEV185595C13] Bernardos R. L. and Raymond P. A. (2006). GFAP transgenic zebrafish. *Gene Expr. Patterns* 6, 1007-1013. 10.1016/j.modgep.2006.04.006 16765104

[DEV185595C14] Bhardwaj R. D., Curtis M. A., Spalding K. L., Buchholz B. A., Fink D., Bjork-Eriksson T., Nordborg C., Gage F. H., Druid H., Eriksson P. S. et al. (2006). Neocortical neurogenesis in humans is restricted to development. *Proc. Natl. Acad. Sci. USA* 103, 12564-12568. 10.1073/pnas.0605177103 16901981 PMC1567918

[DEV185595C15] Blondel V. D., Guillaume J. L., Lambiotte R. and Lefebvre E. (2008). Fast unfolding of communities in large networks. *J. Stat. Mech. Theory Exp*. 2008, 10008 10.1088/1742-5468/2008/10/P10008

[DEV185595C16] Brand M., Granato M. and Nüsslein-Volhard C. (2002). Keeping and raising zebrafish. In *Zebrafish* (ed. Nüsslein-Volhard C. and Dahm R.), pp. 7-37. Oxford, UK: Oxford University Press.

[DEV185595C17] Briggs J. A., Weinreb C., Wagner D. E., Megason S., Peshkin L., Kirschner M. W. and Klein A. M. (2018). The dynamics of gene expression in vertebrate embryogenesis at single-cell resolution. *Science* 360, eaar5780 10.1126/science.aar5780 29700227 PMC6038144

[DEV185595C82] Buettner F. and Theis F. J. (2012). A novel approach for resolving differences in single-cell gene expression patterns from zygote to blastocyst. *Bioinformatics* 28, i626-i632. 10.1093/bioinformatics/bts385 22962491 PMC3436812

[DEV185595C18] Camp J. G., Wollny D. and Treutlein B. (2018). Single-cell genomics to guide human stem cell and tissue engineering. *Nat. Methods* 15, 661-667. 10.1038/s41592-018-0113-0 30171231

[DEV185595C19] Coifman R. R., Lafon S., Lee A. B., Maggioni M., Nadler B., Warner F. and Zucker S. W. (2005). Geometric diffusions as a tool for harmonic analysis and structure definition of data: diffusion maps. *Proc. Natl. Acad. Sci. USA* 102, 7426-7431. 10.1073/pnas.0500334102 15899970 PMC1140422

[DEV185595C20] Cosacak M. I., Bhattarai P., Reinhardt S., Petzold A., Dahl A., Zhang Y. and Kizil C. (2019). Single-cell transcriptomics analyses of neural stem cell heterogeneity and contextual plasticity in a zebrafish brain model of amyloid toxicity. *Cell Rep* 27, 1307-1318.e3. 10.1016/j.celrep.2019.03.090 31018142

[DEV185595C21] Dray N., Bedu S., Vuillemin N., Alunni A., Coolen M., Krecsmarik M., Supatto W., Beaurepaire E. and Bally-Cuif L. (2015). Large-scale live imaging of adult neural stem cells in their endogenous niche. *Development* 142, 3592-3600. 10.1242/dev.123018 26395477 PMC4631764

[DEV185595C83] Edelmann K., Glashauser L., Sprungala S., Hesl B., Fritschle M., Ninkovic J., Godinho L. and Chapouton P. (2013). Increased radial glia quiescence, decreased reactivation upon injury and unaltered neuroblast behavior underlie decreased neurogenesis in the aging zebrafish telencephalon. *J. Comp. Neurol.* 521, 3099-3115. 10.1002/cne.23347 23787922

[DEV185595C22] Ekström P., Johnsson C.-M. and Ohlin L.-M. (2001). Ventricular proliferation zones in the brain of an adult teleost fish and their relation to neuromeres and migration (secondary matrix) zones. *J. Comp. Neurol.* 436, 92-110. 10.1002/cne.1056 11413549

[DEV185595C23] Ernst A. and Frisén J. (2015). Adult neurogenesis in humans- common and unique traits in mammals. *PLoS Biol.* 13, e1002045 10.1371/journal.pbio.1002045 25621867 PMC4306487

[DEV185595C24] Farrell J. A., Wang Y., Riesenfeld S. J., Shekhar K., Regev A. and Schier A. F. (2018). Single-cell reconstruction of developmental trajectories during zebrafish embryogenesis. *Science* 360, eaar3131 10.1126/science.aar3131 29700225 PMC6247916

[DEV185595C25] Fausett B. V. and Goldman D. (2006). A role for alpha1 tubulin-expressing Muller glia in regeneration of the injured zebrafish retina. *J. Neurosci.* 26, 6303-6313. 10.1523/JNEUROSCI.0332-06.2006 16763038 PMC6675181

[DEV185595C26] Ferri R. T. and Levitt P. (1993). Cerebral cortical progenitors are fated to produce region-specific neuronal populations. *Cereb. Cortex* 3, 187-198. 10.1093/cercor/3.3.187 8324369

[DEV185595C27] Frisén J. (2016). Neurogenesis and gliogenesis in nervous system plasticity and repair. *Annu. Rev. Cell Dev. Biol.* 32, 127-141. 10.1146/annurev-cellbio-111315-124953 27298094

[DEV185595C28] Furlan G., Cuccioli V., Vuillemin N., Dirian L., Muntasell A. J., Coolen M., Dray N., Bedu S., Houart C., Beaurepaire E. et al. (2017). Life-long neurogenic activity of individual neural stem cells and continuous growth establish an outside-in architecture in the teleost pallium. *Curr. Biol.* 27, 3288-3301.e3283. 10.1016/j.cub.2017.09.052 29107546 PMC5678050

[DEV185595C29] Ganz J. and Brand M. (2016). Adult neurogenesis in fish. *Cold Spring Harb. Perspect Biol.* 10.1101/cshperspect.a019018 PMC4930922 26747664

[DEV185595C30] Ganz J., Kaslin J., Hochmann S., Freudenreich D. and Brand M. (2010). Heterogeneity and Fgf dependence of adult neural progenitors in the zebrafish telencephalon. *Glia* 58, 1345-1363. 10.1002/glia.21012 20607866

[DEV185595C31] Ganz J., Kaslin J., Freudenreich D., Machate A., Geffarth M. and Brand M. (2012). Subdivisions of the adult zebrafish subpallium by molecular marker analysis. *J. Comp. Neurol.* 520, 633-655. 10.1002/cne.22757 21858823

[DEV185595C32] Gerber T., Murawala P., Knapp D., Masselink W., Schuez M., Hermann S., Gac- Santel M., Nowoshilow S., Kageyama J., Khattak S. et al. (2018). Single- cell analysis uncovers convergence of cell identities during axolotl limb regeneration. *Science* 362, eaaq0681 10.1126/science.aaq0681 30262634 PMC6669047

[DEV185595C33] Grandel H. and Brand M. (2013). Comparative aspects of adult neural stem cell activity in vertebrates. *Dev. Genes Evol.* 223, 131-147. 10.1007/s00427-012-0425-5 23179636

[DEV185595C34] Grandel H., Kaslin J., Ganz J., Wenzel I. and Brand M. (2006). Neural stem cells and neurogenesis in the adult zebrafish brain: origin, proliferation dynamics, migration and cell fate. *Dev. Biol.* 295, 263-277. 10.1016/j.ydbio.2006.03.040 16682018

[DEV185595C35] Haghverdi L., Buettner F. and Theis F. J. (2015). Diffusion maps for high- dimensional single-cell analysis of differentiation data. *Bioinformatics* 31, 2989-2998. 10.1093/bioinformatics/btv325 26002886

[DEV185595C36] Hiraiwa A., Fujita M., Nagasaka T., Adachi A., Ohashi M. and Ishibashi M. (1997). Immunolocalization of hCDC47 protein in normal and neoplastic human tissues and its relation to growth. *Int. J. Cancer* 74, 180-184. 10.1002/(SICI)1097-0215(19970422)74:2<180::AID-IJC7>3.0.CO;2-V 9133452

[DEV185595C37] Hochgerner H., Zeisel A., Lönnerberg P. and Linnarsson S. (2018). Conserved properties of dentate gyrus neurogenesis across postnatal development revealed by single-cell RNA sequencing. *Nat. Neurosci.* 21, 290-299. 10.1038/s41593-017-0056-2 29335606

[DEV185595C38] Huttner H. B., Bergmann O., Salehpour M., Rácz A., Tatarishvili J., Lindgren E., Csonka T., Csiba L., Hortobágyi T., Méhes G. et al. (2014). The age and genomic integrity of neurons after cortical stroke in humans. *Nat. Neurosci.* 17, 801-803. 10.1038/nn.3706 24747576

[DEV185595C39] Kaslin J., Ganz J. and Brand M. (2008). Proliferation, neurogenesis and regeneration in the non-mammalian vertebrate brain. *Philos. Trans. R. Soc. Lond. B Biol. Sci.* 363, 101-122. 10.1098/rstb.2006.2015 17282988 PMC2605489

[DEV185595C40] Kaslin J., Kroehne V., Ganz J., Hans S. and Brand M. (2017). Distinct roles of neuroepithelial-like and radial glia-like progenitor cells in cerebellar regeneration. *Development* 144, 1462-1471. 10.1242/dev.144907 28289134

[DEV185595C81] Kawakami K., Takeda H., Kawakami N., Kobayashi M., Matsuda N. and Mishina M. (2004). A transposon-mediated gene trap approach identifies developmentally regulated genes in zebrafish. *Dev. Cell* 7, 133-144. 10.1016/j.devcel.2004.06.005 15239961

[DEV185595C41] Kesavan G., Chekuru A., Machate A. and Brand M. (2017). CRISPR/Cas9- mediated zebrafish knock-in as a novel strategy to study midbrain-hindbrain boundary development. *Front. Neuroanat.* 11, 52 10.3389/fnana.2017.00052 28713249 PMC5492657

[DEV185595C42] Kesavan G., Hammer J., Hans S. and Brand M. (2018). Targeted knock-in of CreER T2 in zebrafish using CRISPR/Cas9. *Cell Tissue Res.* 372, 41-50. 10.1007/s00441-018-2798-x 29435650

[DEV185595C43] Kester L. and van Oudenaarden A. (2018). Single-cell transcriptomics meets lineage tracing. *Cell Stem Cell* 23, 166-179. 10.1016/j.stem.2018.04.014 29754780

[DEV185595C44] KrastevaV., BuscarletM., Diaz-TellezA., BernardM.-A., CrabtreeG. R. and LessardJ. A. (2012). The BAF53a subunit of SWI/SNF-like BAF complexes is essential for hemopoietic stem cell function. *Blood* 120, 4720-4732. 10.1182/blood-2012-04-42704723018638

[DEV185595C45] Kroehne V., Kaslin J. and Brand M. (2009). 19-P003 Proliferation and cell fates during regeneration of the adult zebrafish brain. *Mech. Dev.* 126, 291-292. 10.1016/j.mod.2009.06.791

[DEV185595C46] Kroehne V., Freudenreich D., Hans S., Kaslin J. and Brand M. (2011). Regeneration of the adult zebrafish brain from neurogenic radial glia-type progenitors. *Development* 138, 4831-4841. 10.1242/dev.072587 22007133

[DEV185595C47] Kroehne V., Tsata V., Marrone L., Froeb C., Reinhardt S., Gompf A., Dahl A., Sterneckert J. and Reimer M. M. (2017). Primary spinal OPC culture system from adult zebrafish to study oligodendrocyte differentiation in vitro. *Front. Cell Neurosci.* 11, 284 10.3389/fncel.2017.00284 28959189 PMC5603699

[DEV185595C48] Kunze A., Congreso M. R., Hartmann C., Wallraff-Beck A., Huttmann K., Bedner P., Requardt R., Seifert G., Redecker C., Willecke K. et al. (2009). Connexin expression by radial glia-like cells is required for neurogenesis in the adult dentate gyrus. *Proc. Natl. Acad. Sci. USA* 106, 11336-11341. 10.1073/pnas.0813160106 19549869 PMC2700144

[DEV185595C49] Levine J. H., Simonds E. F., Bendall S. C., Davis K. L., Amir E.-D., Tadmor M. D., Litvin O., Fienberg H. G., Jager A., Zunder E. R. et al. (2015). Data-driven phenotypic dissection of AML reveals progenitor-like cells that correlate with prognosis. *Cell* 162, 184-197. 10.1016/j.cell.2015.05.047 26095251 PMC4508757

[DEV185595C50] Li X., Zhao X., Fang Y., Jiang X., Duong T., Fan C., Huang C.-C. and Kain S. R. (1998). Generation of destabilized green fluorescent protein as a transcription reporter. *J. Biol. Chem.* 273, 34970-34975. 10.1074/jbc.273.52.34970 9857028

[DEV185595C51] Lun A. T., McCarthy D. J. and Marioni J. C. (2016). A step-by-step workflow for low-level analysis of single-cell RNA-seq data with Bioconductor. *F1000Res* 5, 2122 10.12688/f1000research.9501.2 27909575 PMC5112579

[DEV185595C52] Maaten L. V. d. and Hinton G. (2008). Visualizing data using t-SNE. *J. Mach. Learn. Res.* 9, 2579-2605.

[DEV185595C53] März M., Chapouton P., Diotel N., Vaillant C., Hesl B., Takamiya M., Lam C. S., Kah O., Bally-Cuif L. and Strahle U. (2010a). Heterogeneity in progenitor cell subtypes in the ventricular zone of the zebrafish adult telencephalon. *Glia* 58, 870-888. 10.1002/glia.20971 20155821

[DEV185595C54] März M., Schmidt R., Rastegar S. and Strahle U. (2010b). Expression of the transcription factor Olig2 in proliferating cells in the adult zebrafish telencephalon. *Dev. Dyn.* 239, 3336-3349. 10.1002/dvdy.22455 20981834

[DEV185595C55] März M., Schmidt R., Rastegar S. and Strahle U. (2011). Regenerative response following stab injury in the adult zebrafish telencephalon. *Dev. Dyn.* 240, 2221-2231. 10.1002/dvdy.22710 22016188

[DEV185595C56] McCarthy D. J., Campbell K. R., Lun A. T. and Wills Q. F. (2017). Scater: pre- processing, quality control, normalization and visualization of single-cell RNA- seq data in R. *Bioinformatics* 33, 1179-1186. 10.1093/bioinformatics/btw777 28088763 PMC5408845

[DEV185595C57] McInnes L. and Healy J. (2018). UMAP: uniform manifold approximation and projection for dimension reduction. *arXiv:1802.03426 [stat,ML]*.

[DEV185595C58] Mueller T. and Guo S. (2009). The distribution of GAD67-mRNA in the adult zebrafish (teleost) forebrain reveals a prosomeric pattern and suggests previously unidentified homologies to tetrapods. *J. Comp. Neurol.* 516, 553-568. 10.1002/cne.22122 19673006 PMC2828780

[DEV185595C59] Ohab J. J., Fleming S., Blesch A. and Carmichael S. T. (2006). A neurovascular niche for neurogenesis after stroke. *J. Neurosci.* 26, 13007-13016. 10.1523/JNEUROSCI.4323-06.2006 17167090 PMC6674957

[DEV185595C84] Ohtani K., Iwanaga R., Nakamura M., Ikeda Ikeda, Yabuta N., Tsuruga H., and Nojima H. (1999). Cell growth-regulated expression of mammalian MCM5 and MCM6 genes mediated by the transcription factor E2F. *Oncogene* 18, 2299-2309. 10.1038/sj.onc.1202544 10327050

[DEV185595C60] Park H.-C., Kim C.-H., Bae Y.-K., Yeo S.-Y., Kim S.-H., Hong S.-K., Shin J., Yoo K.-W., Hibi M., Hirano T. et al. (2000). Analysis of upstream elements in the HuC promoter leads to the establishment of transgenic zebrafish with fluorescent neurons. *Dev. Biol.* 227, 279-293. 10.1006/dbio.2000.9898 11071755

[DEV185595C61] Park H.-C., Shin J., Roberts R. K. and Appel B. (2007). An olig2 reporter gene marks oligodendrocyte precursors in the postembryonic spinal cord of zebrafish. *Dev. Dyn.* 236, 3402-3407. 10.1002/dvdy.21365 17969181

[DEV185595C62] Picelli S., Faridani O. R., Björklund A. K., Winberg G., Sagasser S. and Sandberg R. (2014). Full-length RNA-seq from single cells using Smart-seq2. *Nat. Protoc.* 9, 171-181. 10.1038/nprot.2014.006 24385147

[DEV185595C63] Raj B., Wagner D. E., McKenna A., Pandey S., Klein A. M., Shendure J., Gagnon J. A. and Schier A. F. (2018). Simultaneous single-cell profiling of lineages and cell types in the vertebrate brain. *Nat. Biotechnol.* 36, 442-450. 10.1038/nbt.4103 29608178 PMC5938111

[DEV185595C64] Reimer M. M., Sorensen I., Kuscha V., Frank R. E., Liu C., Becker C. G. and Becker T. (2008). Motor neuron regeneration in adult zebrafish. *J. Neurosci.* 28, 8510-8516. 10.1523/JNEUROSCI.1189-08.2008 18716209 PMC6671064

[DEV185595C65] Rodriguez Viales R., Diotel N., Ferg M., Armant O., Eich J., Alunni A., März M., Bally-Cuif L., Rastegar S. and Strähle U. (2015). The helix-loop-helix protein id1 controls stem cell proliferation during regenerative neurogenesis in the adult zebrafish telencephalon. *Stem Cells* 33, 892-903. 10.1002/stem.1883 25376791

[DEV185595C66] Rothenaigner I., Krecsmarik M., Hayes J. A., Bahn B., Lepier A., Fortin G., Gotz M., Jagasia R. and Bally-Cuif L. (2011). Clonal analysis by distinct viral vectors identifies bona fide neural stem cells in the adult zebrafish telencephalon and characterizes their division properties and fate. *Development* 138, 1459-1469. 10.1242/dev.058156 21367818

[DEV185595C67] Saha B., Peron S., Murray K., Jaber M. and Gaillard A. (2013). Cortical lesion stimulates adult subventricular zone neural progenitor cell proliferation and migration to the site of injury. *Stem Cell Res.* 11, 965-977. 10.1016/j.scr.2013.06.006 23900166

[DEV185595C68] Shin J., Park H.-C., Topczewska J. M., Mawdsley D. J. and Appel B. (2003). Neural cell fate analysis in zebrafish using olig2 BAC transgenics. *Methods Cell Sci.* 25, 7-14. 10.1023/B:MICS.0000006847.09037.3a 14739582

[DEV185595C69] Skaggs K., Goldman D. and Parent J. M. (2014). Excitotoxic brain injury in adult zebrafish stimulates neurogenesis and long-distance neuronal integration. *Glia* 62, 2061-2079. 10.1002/glia.22726 25043622 PMC4205181

[DEV185595C85] Sobecki M., Mrouj K., Colinge J., Gerbe F., Jay P., Krasinska L., Dulic V. and Fisher D. (2017). Cell-cycle regulation accounts for variability in Ki-67 expression levels. *Cancer Res.* 77, 2722-2734. 10.1158/0008-5472.CAN-16-0707 28283655

[DEV185595C70] Spanjaard B., Hu B., Mitic N., Olivares-Chauvet P., Janjuha S., Ninov N. and Junker J. P. (2018). Simultaneous lineage tracing and cell-type identification using CRISPR-Cas9-induced genetic scars. *Nat. Biotechnol.* 36, 469-473. 10.1038/nbt.4124 29644996 PMC5942543

[DEV185595C71] Traag V. A. (2015). louvain-igraph: v0.5.3. Zenodo.

[DEV185595C72] Tsata V., Kroehne V., Reinhardt S., El-Armouche A., Brand M., Wagner M. and Reimer M. M. (2019). Electrophysiological properties of adult zebrafish oligodendrocyte progenitor cells. *Front. Cell Neurosci.* 13, 102 10.3389/fncel.2019.00102 31031593 PMC6473327

[DEV185595C73] Turner K. J., Hawkins T. A., Yáñez J., Anadón R., Wilson S. W. and Folgueira M. (2016). Afferent connectivity of the zebrafish habenulae. *Front. Neural Circuits* 10, 30 10.3389/fncir.2016.00030 27199671 PMC4844923

[DEV185595C74] Ulyanov D. (2018). Multicore-TSNE. https://github.com/DmitryUlyanov/Multicore-TSNE.

[DEV185595C75] Wagner D. E., Weinreb C., Collins Z. M., Briggs J. A., Megason S. G. and Klein A. M. (2018). Single-cell mapping of gene expression landscapes and lineage in the zebrafish embryo. *Science* 360, 981-987. 10.1126/science.aar4362 29700229 PMC6083445

[DEV185595C76] Wolf F. A., Angerer P. and Theis F. J. (2018). SCANPY: large-scale single-cell gene expression data analysis. *Genome Biol.* 19, 15 10.1186/s13059-017-1382-0 29409532 PMC5802054

[DEV185595C77] Wolf F. A., Hamey F. K., Plass M., Solana J., Dahlin J. S., Göttgens B., Rajewsky N., Simon L. and Theis F. J. (2019). PAGA: graph abstraction reconciles clustering with trajectory inference through a topology preserving map of single cells. *Genome Biol.* 20, 59 10.1186/s13059-019-1663-x 30890159 PMC6425583

[DEV185595C78] Yu S. and He J. (2019). Stochastic cell-cycle entry and cell-state-dependent fate outputs of injury-reactivated tectal radial glia in zebrafish. *eLife* 8, e48660 10.7554/eLife.48660 31442201 PMC6707787

[DEV185595C79] Zhong L. and Gerges N. Z. (2012). Neurogranin targets calmodulin and lowers the threshold for the induction of long-term potentiation. *PLoS ONE* 7, e41275 10.1371/journal.pone.0041275 22848456 PMC3405117

[DEV185595C80] Zupanc G. K. H., Hinsch K. and Gage F. H. (2005). Proliferation, migration, neuronal differentiation, and long-term survival of new cells in the adult zebrafish brain. *J. Comp. Neurol.* 488, 290-319. 10.1002/cne.20571 15952170

