# Supplementary material for: Single cell sequencing of radial glia progeny reveals the diversity of newborn neurons in the adult zebrafish brain
Source: Development. 2020 Jan 9;147(1):dev185595. doi: 10.1242/dev.185595 (PMC6983714; doi:10.1242/dev.185595)
Supplement: Supplementary information [file develop-147-185595-s1.pdf]

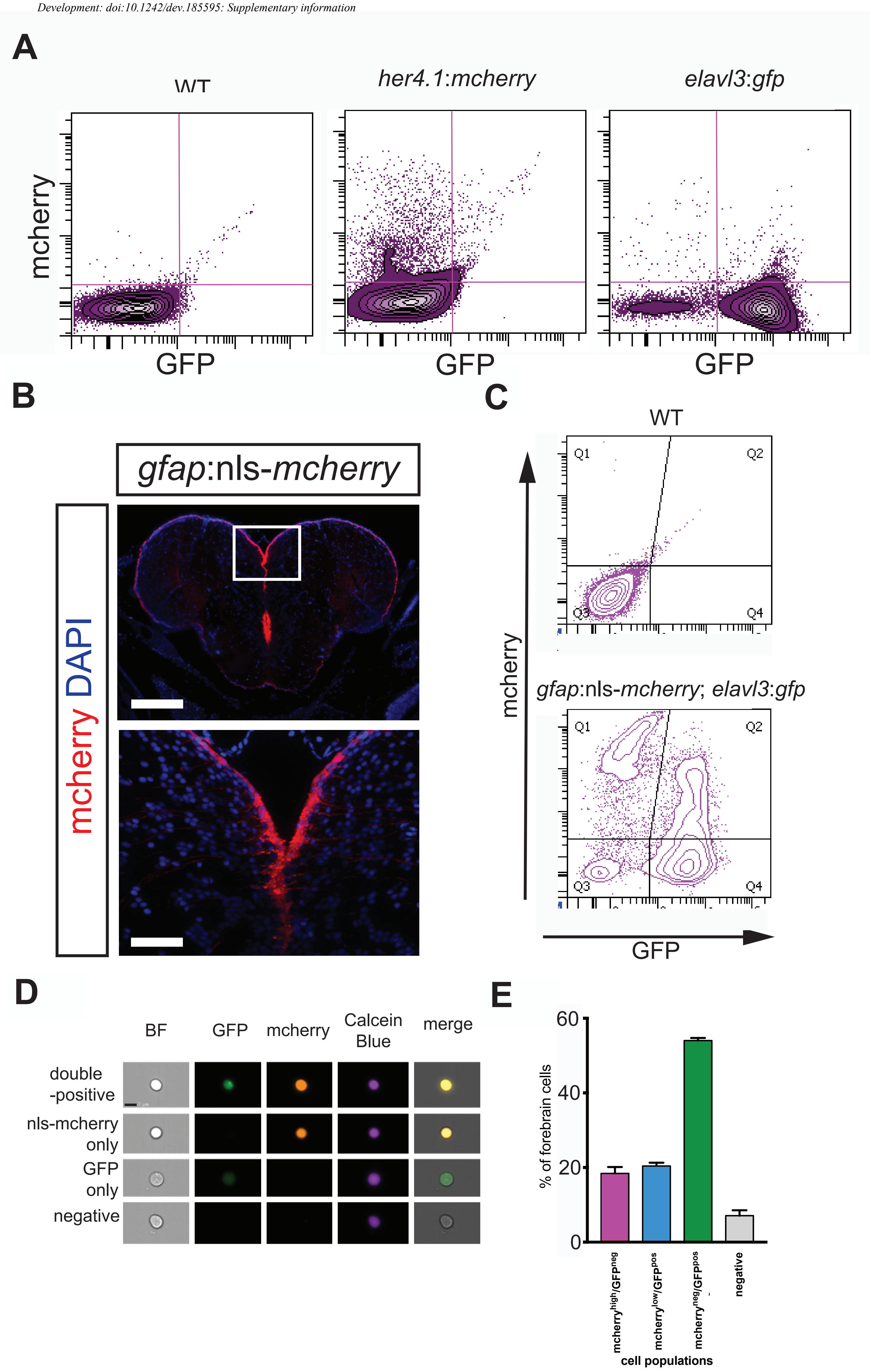
A
WT
her4.1:mcherry
elavl3:gfp
mcherry
GFP
GFP
GFP
B
gfap:nls-mcherry
mcherry DAPI
C
WT
Q1
Q2
Q3
Q4
mcherry
gfap:nls-mcherry; elavl3:gfp
Q1
Q2
Q3
Q4
GFP
D
BF
GFP
mcherry
Calcein Blue
merge
double -positive
nls-mcherry only
GFP only
negative
E
60
40
20
0
% of forebrain cells
mcherryhigh/GFPneg
mcherrylow/GFPpos
mcherryneg/GFPpos
negative
cell populations

Figure S1

Figure S1

(A) Representative FACS-plots showing forebrain cells from WT (left) or single transgenic *her4.1*:*mcherry* (middle) and *elavl3:gfp* reporter fish (right), analyzed for mcherry- (y-axis) and GFP-fluorescence (x-axis). (B) Optical section of immunostaining for mcherry in *gfap*:nls-*mcherry* reporter fish, showing expression of the mcherry reporter in RG. Nuclei are stained with DAPI.The lower image shows a magnification of the boxed area of the top panel (C) Representative FACS-plots showing forebrain cells from WT (top) and *gfap*:nls-*mcherry*;*elavl3:gfp* double reporter fish (bottom). Note the clearly separated populations of mcherry^{high}/GFP^{neg}, mcherry^{low}/GFP^{pos} and mcherry^{neg}/GFP^{pos} cells. (D) Representative single cells imaged in flow cytometry for mcherry^{pos}/GFP^{pos} (top), mcherry^{pos}/GFP^{neg} (middle, top), mcherry^{neg}/GFP^{pos} (middle,bottom) and , mcherry^{neg}/GFP^{neg} (bottom). At least 20 cells were analyzed and no doublets were seen, excluding that double-positive cells represent doublets. (E) Quantification of cells in the 4 quadrants shown in (B) indicating that mcherry^{low}/GFP^{pos} NBNs can be found in a frequency comparable to mcherry^{high}/GFP^{neg} RGs. N=3; data are presented as mean±SEM. Scale bar = 200µm (B,top), 30µm (B,bottom) or 7 µm (D).

Development • Supplementary information

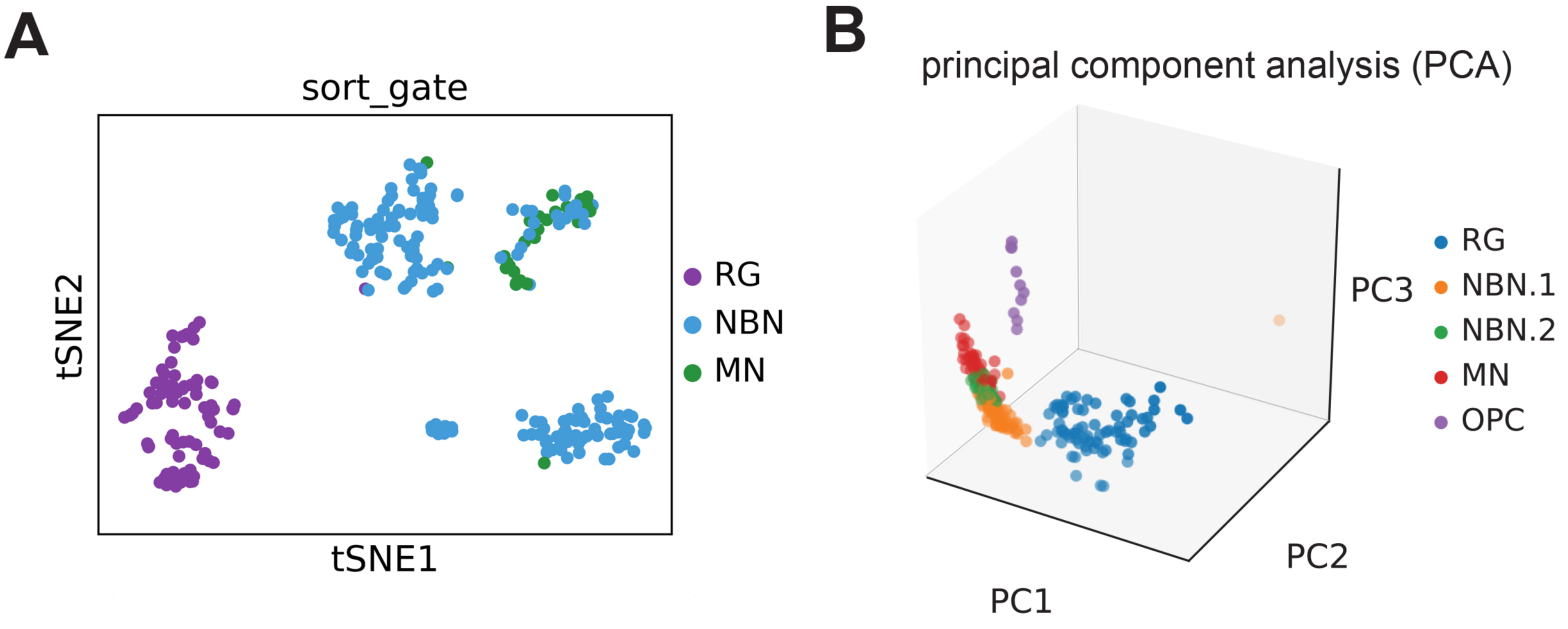

Figure S2

Figure S2

(A) tSNE plot showing the distribution of sorted cell types according to FACS gates (RG (purple), NBNs (cyan), MNs (green)) within the 5 transcriptome-based clusters shown in Figure 2B. The data indicate that RG and MNs each contribute to only one cluster, while NBN are heterogeneous and a subset also forms a common cluster with MNs (B) Plot for dimensionality reduction using principal component analysis (PCA), resulting in similar cluster as those generated with tSNE.

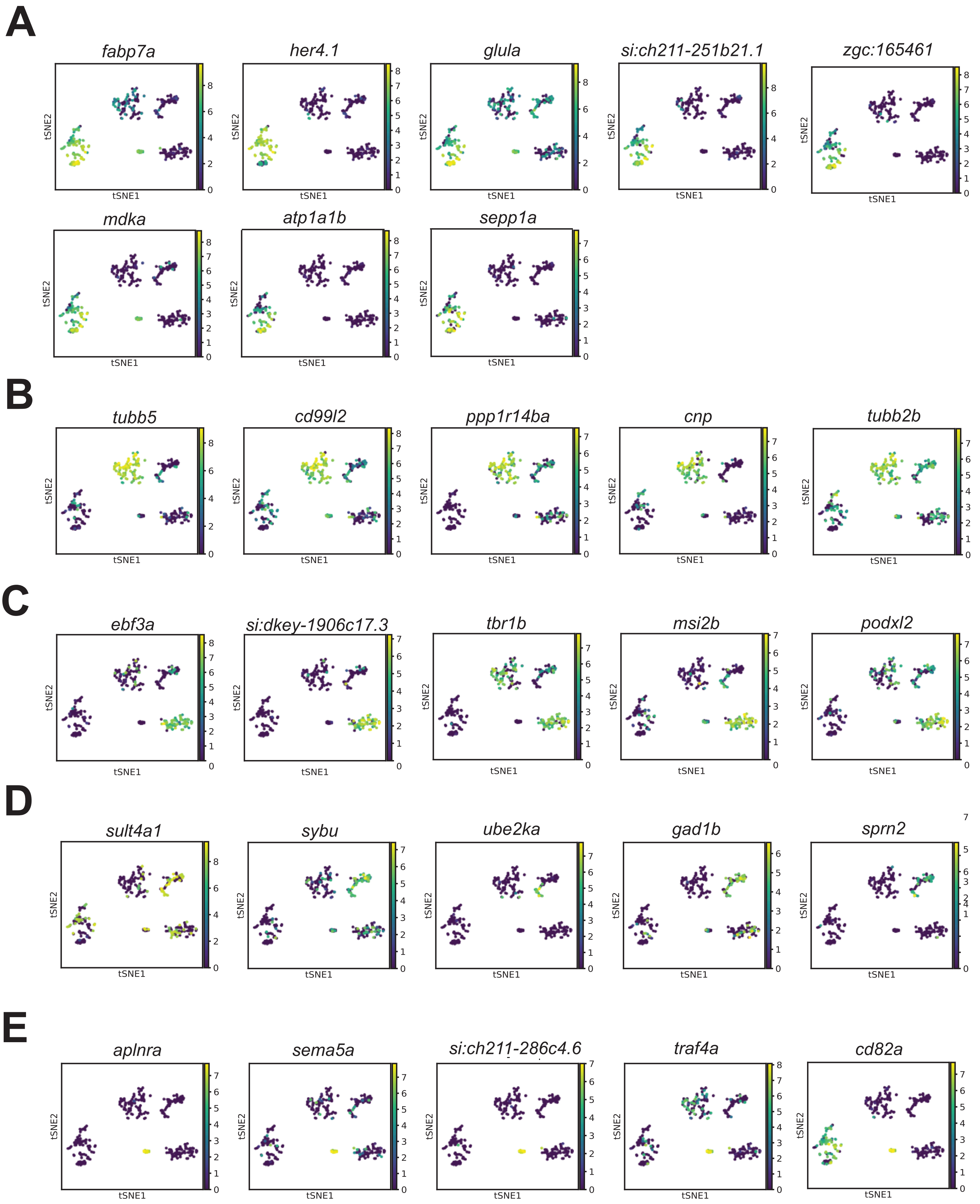

Figure S3

Figure S3

(A) Expression of RG marker genes (*fabp7a*, *her4.1*, *glula*, *si:ch211-251b21.1*, *zgc:165461*, *atp1a1b*, *sepp1a*, *mdka*) in t-SNE plots. Each t-SNE plot consists of n=264 cells. Cells in each plot are colored by their expression of each marker gene according to the adjacent scale. (B) Expression of NBN.1 marker genes (*tubb5*, *cd99l2*, *cnp, ppp1r14ba, tuba2a*) in t-SNE plots as in (A). (C) Expression of NBN.2 marker genes (*ebf3a*, *si:dkey-106c17.3*, *tbr1b*, *msi2b, podxl2*) in t-SNE plots as in (A). (D) Expression of MN marker genes (*sult4a1, sybu*, ube2ka, *gad1b*, *sprn2*) in t-SNE plots as in (A). (E) Expression of OPC marker genes (*aplnra*, *sema5a, si:ch211-286c4.6,* ***traf4a,*** *cd82a*) in t-SNE plots as in (A).

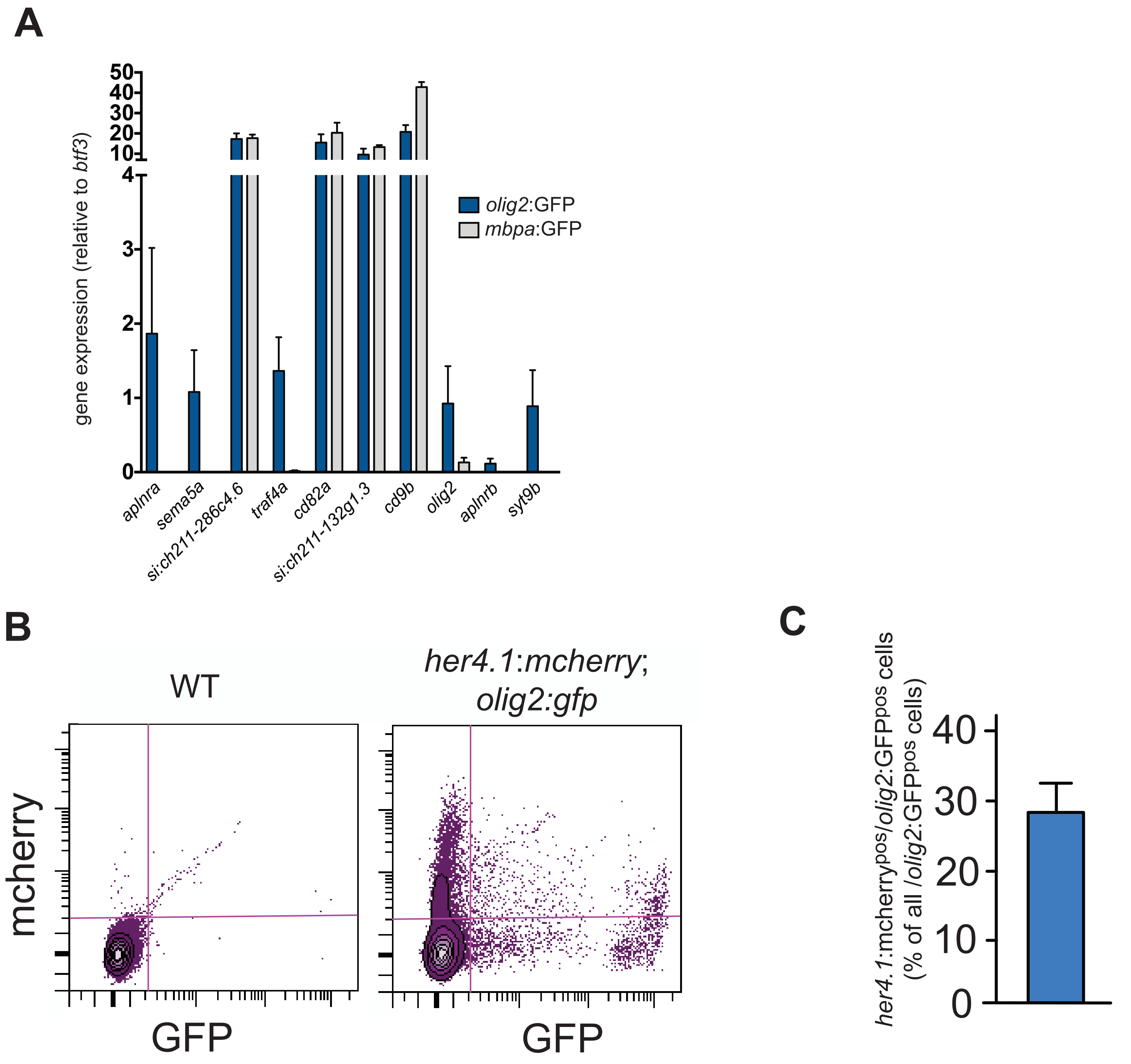

Figure S4

Figure S4

(A) Expression of top 10 OPC marker genes, identified in this study (*aplnra*, *sema5a, si:ch211-286c4.6***,** *traf4a***,** *cd82a*, *si:ch211132g1.3, cd9b, olig2, aplnrb, syt9b*) in sorted OPCs (*olig2*:GFP^{+}, blue bars) or sorted oligodendrocytes (*mbpa*:GFP+, grey bars) from the adult zebrafish spinal cord. A subset of OPC markers (*aplnra*, *sema5a, traf4a olig2, aplnrb, syt9b*) was specifically expressed in OPC, while *si:ch211-286c4.6, cd82a*, *si:ch211132g1.3 and* cd9b were consistently expressed in the oligodendrocyte lineage. Data are taken from Kroehne et al. (2017). (B) Representative FACS-plots showing forebrain cells from WT (left) or *her4.1*:*mcherry*; *olig2*:*gfp* double-transgenic reporters (right), analyzed for mcherry- (y-axis) and GFP-fluorescence (x-axis). Note the substantial proportion of *her4.1*:mcherry^{pos}/ *olig2*:GFP^{pos} cells. (C) Quantification of *her4.1*:mcherry^{pos}/ *olig2*:GFP^{pos} cells as fraction of all *olig2*:GFP^{pos} cells. N=3; data are presented as mean±SEM.

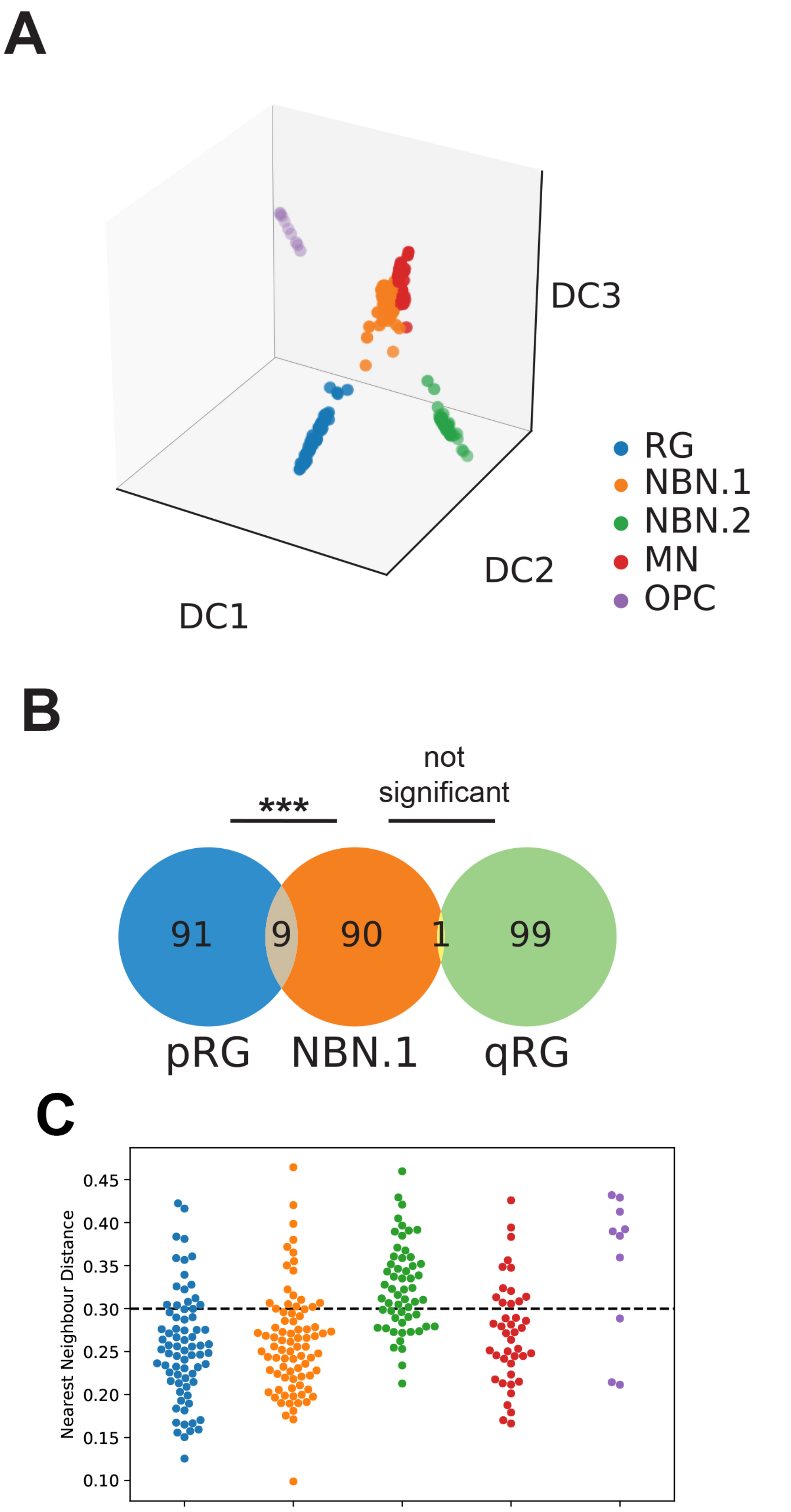

Figure S5

Figure S5

(A) Lineage trajectory analysis of single-cell RNAseq data from RG, NBNs, MNs and OPCs from the adult zebrafish forebrain as shown in Figure 3A, but including OPCs. Color coding corresponds to the different cell clusters identified in Figure 2B. The OPCs are transcriptionally disconnected from the RG and their neuronal progeny. (B) Venn diagram showing overlapping expression of the top 100 marker genes for NBN.1 cells (middle, orange) with the top 100 marker genes of proliferating RG (pRG,left, blue) or quiescent RG (qRG, right, green). Significant statistical overrepresentation of NBN.1 marker genes is found in pRG (9 co-expressed NBN.1 marker genes), but not in qRG (1 co-expressed NBN.1 marker gene). ***=p<0.0001 (C) Diagram of nearest neighbor distance for RG (blue), NBN.1 cells (orange), NBN.2 cells (green), MNs (red) and OPCs (purple) to their most similar mammalian cells from Hochgerner et al. (2018). Each dot represents one cell. A dashed line indicates the cut-off for the 66 percent quantile. Cells within the 66 percent quantile were included into the analysis of corresponding murine cell type heterogeneity (Fig. 4C,D).

Development • Supplementary information
